# Proton Exchange Membrane with Dual‐Active‐Center Surpasses the Conventional Temperature Limitations of Fuel Cells

**DOI:** 10.1002/advs.202417259

**Published:** 2025-01-21

**Authors:** Yucong Liao, Shengqiu Zhao, Rui wang, Junjie Zhang, Hao Li, Bingxuan Liu, Yao Li, Aojie Zhang, Tian Tian, Haolin Tang

**Affiliations:** ^1^ State Key Laboratory of Advanced Technology for Materials Synthesis and Processing Wuhan University of Technology Wuhan 430070 P. R. China; ^2^ National energy key laboratory for new hydrogen‐ammonia energy technologies Foshan Xianhu Laboratory Foshan 528200 P. R. China; ^3^ Hubei Key Laboratory of Fuel Cell Wuhan 430070 P. R. China

**Keywords:** dual active centers, high temperature and low humidity, hydrophilic nanodomains, perfluoroimide multi‐acid ionomers, proton exchange membrane

## Abstract

High temperature‐proton exchange membrane fuel cells (HT‐PEMFC) call for ionomers with low humidity dependence and elevated‐temperature resistance. Traditional perfluorosulfonic acid (PFSA) ionomers encounter challenges in meeting these stringent requirements. Herein, this study reports a perfluoroimide multi‐acid (PFMA) ionomer with dual active centers achieved through the incorporation of sulfonimide and phosphonic acid groups into the side chain. The fluorocarbon skeleton and multi‐acid side chain structure facilitate the segregation of hydrophilic and hydrophobic microphases, augmenting the short‐range ordering of hydrophilic nanodomains. Furthermore, the introduction of a rigid segment‐benzene ring is employed to decrease side chain flexibility and raise the glass transition temperature. Notably, the prepared membrane exhibits a conductivity of 41 mS cm^−1^ at 40% relative humidity, showcasing a 1.8 times improvement over that of PFSA. Additionally, the power output of the H_2_‐air fuel cell based on this membrane reaches 1.5 W cm^−2^ at 105 °C, marking a substantial 2.3 times enhancement compared to the PFSA. This work demonstrates the unique advantages of perfluorinated ionomers with multiple protogenic groups in the development of high‐performance high‐temperature electrolyte materials.

## Graphical abstract

Perfluoroimide multi‐acid (PFMA) ionomer with dual active centers is developed by incorporating sulfonimide and phosphonic acid groups. The fluorocarbon backbone and multi‐acid side chain structure promote the segregation of hydrophilic and hydrophobic microphases, constructed a unique proton transport channel. The benzene ring as a rigid segment, is utilized to reduce side chain flexibility and elevate the glass transition temperature.

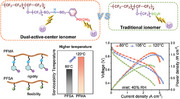

## 1 Introduction

The proton exchange membrane fuel cell (PEMFC) stands as an efficient device for converting hydrogen into electricity.^[^ ^1^, ^2^, ^3^ ^]^ However, traditional perfluorosulfonic acid (PFSA) proton exchange membrane (PEM) is limited to operating at relatively low temperatures, ≈80 °C.^[^ ^4^ ^]^ This membrane, characterized by a low glass transition temperature (<120 °C) and a significant dependence on moisture, exhibits a notable reduction in proton conductivity under low humidity conditions.^[^ ^5^, ^6^ ^]^ The immediate imperative lies in elevating the operational temperature of fuel cells, a measure that can effectively alleviate flooding issues and optimize the humidification system.^[^ ^7^ ^]^ The New Energy and Industrial Technology Development Organization in Japan has established ambitious targets to increase fuel cell operating temperatures to 105 °C by 2030 and 120 °C by 2040. Additionally, As the 2025 objective outlined by the U.S. Department of Energy, the proton exchange membrane should possess a low area‐specific resistance (ASR) of 0.02 ohm cm^2^ under the specified operating conditions of high temperature (120 °C) and low relative humidity.^[^ ^8^, ^9^ ^]^

Most proton exchange membranes require high hydration levels to ensure proton conductivity, limiting operation to temperatures below the boiling point of water (100 °C).^[^ ^10^, ^11^ ^]^ Consequently, endeavors have been undertaken to improve the proton conductivity at lower hydration levels, which involve the development of ionomers incorporating diverse acidic functional groups.^[^ ^12^, ^13^, ^14^, ^15^, ^16^, ^17^, ^18^ ^]^ In addition to sulfonic acid groups, other protogenic groups serve as proton conduction sites, such as carboxylic,^[^ ^19^ ^]^ phosphonic,^[^ ^20^ ^]^ and sulfonimide functional groups.^[^ ^21^ ^]^ The amphoteric nature of the phosphonic acid group enables it to function both as a proton donor and acceptor concurrently, facilitating proton migration through the hydrogen bond network via the Grotthuss mechanism under conditions of elevated temperature and low humidity. For instance, Patrick Jannasch et al. introduced phosphinated aromatic polymers featuring highly acidic perfluorophenylphosphonic acid, exhibiting high proton conductivity (130 mS cm^−1^ at 120 °C).^[^ ^22^ ^]^ Various types of phosphinated polymers have been successfully documented, including phosphoric acid porous organic polymers,^[^ ^23^, ^24^ ^]^ phosphinated PBI,^[^ ^25^ ^]^ phosphinated polypentafluorostyrene,^[^ ^26^ ^]^ among others.^[^ ^27^, ^28^ ^]^ Additionally, Sulfonyl imide groups with superacidic properties are also regarded as potential candidates for application in PEMs. Studies by Sumner and colleagues revealed that ionomers containing bis[(perfluoroalkyl)sulfonyl]imide functional groups exhibited slightly superior conductivity compared to Nafion 117.^[^ ^29^, ^30^ ^]^ However, these developed materials are unable to fulfill the need for stable fuel cell operation under the challenging conditions of high temperature and low humidity.

Increasing the number of acidic functional groups is an effective strategy to improve ion exchange capacity.^[^ ^31^ ^]^ Nevertheless, reducing the Equivalent Weight (EW) value leads to decreased crystallinity and mechanical strength. Traditional PFSA membranes typically have a lower threshold of ≈700 g mol^−1^, below which there is a risk of solubility in water. Maintaining backbone crystallinity while reducing the EW value is the optimal solution to this challenge.^[^ ^32^ ^]^ In particular, 3 M company has developed an ionomer (PFIA) with dual acidic functional groups. This side‐chain extension technique enhances the acid content while preserving the polymer backbone.^[^ ^33^ ^]^

**Figure 1:**
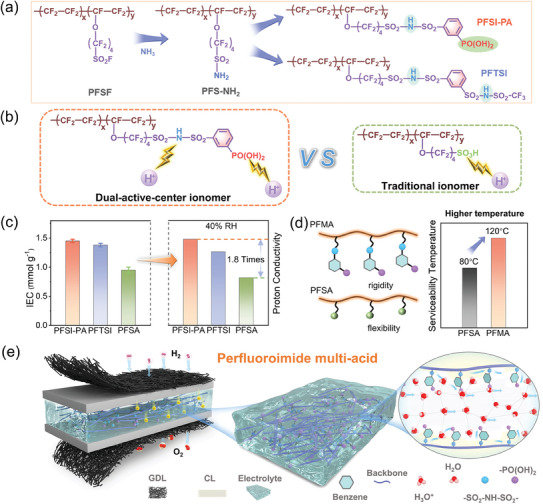
a) The schematic diagram of the synthesis process of PFSI‐PA and PFTSI. b) Structure comparison of the Dual‐active center ionomer (PFSI‐PA) and Traditional ionomer (PFSA). c) The comparison of IEC and proton conductivity for PFSI‐PA, PFTSI and PFSA. d) The schematic diagram compares the side chains of PFMA and PFSA, along with a comparison of their service temperatures. e) The schematic diagram of the PFSI‐PA membrane electrode and the transport process of protons in the membrane.

In this study, we have undertaken the modification of the side chains of perfluoroalkylsulfonyl fluoride (PFSF), a precursor of perfluorosulfonic acid ionomers, culminating in the synthesis of two perfluoroimide multi‐acid (PFMA) ionomers (**Figure** **1**a). These ionomers feature multiple protogenic acid groups in the side chains, including sulfonyl imide and phosphonic acid groups. This structure shares the same backbone as the original PFSA, while the count of acidic functional groups on the side chains has doubled, leading to a reduction in the equivalent weight (Figure 1b). Furthermore, the incorporation of a rigid benzene ring fragment was implemented to diminish side chain flexibility and elevate the glass transition temperature of the ionomer, consequently expanding the operational temperature range of the proton exchange membrane (Figure 1d). The correlation between the proton conductivity of the as‐prepared membranes and water transport behavior was investigated through dynamic vapor adsorption and in‐situ Fourier Transform Infrared spectroscopy testing. Small angle scattering tests were employed to examine the impacts of phase separation structure and hydrophilic channels on proton transport. Through a combination of Density Functional Theory Calculations (DFT) and Molecular Dynamics (MD) simulations, an in‐depth exploration was conducted on the relationship between the charge distribution of PFMA and proton ionization, along with the morphology of the hydration domain, spatial distribution, and diffusion behavior of water molecules and hydrated protons. Comparative analysis with monoacid‐based PFSA revealed that PFMA displayed significantly enhanced water absorption and proton conductivity, facilitating the formation of larger water clusters and hydrophilic channels. Even under low humidity conditions, its proton transport capacity is 1.8 times greater than that of PFSA, resulting in a substantial improvement in the performance of fuel cells utilizing this material. Hence, the investigation of proton exchange membranes with multiple protogenic acid groups holds significant importance for the advancement of crucial membrane materials designed for high‐temperature fuel cells.

## 2 Results and Discussion

### 2.1 Synthesis and Characterization of Samples

The precursor of PFSA, Pfluoroalkylsulfonyl fluoride (PFSF), were reacted with liquid ammonia and yielded a sulfonamide moiety (‐SO_2_‐NH_2_), followed by modifying the chemical structure of Perfluoroalkanesulfonylamides (PFS‐NH_2_) and grafting various protogenic acid groups. As shown in Figure 1a, two perfluoroimide multi‐acid side‐chain ionomers with dual active centers were successfully synthesized. One is perfluorosulfonimide phosphonic acid (PFSI‐PA) containing sulfonimide and phosphonic acid groups, and the other is perfluorodisulfonimide (PFTSI) containing two sulfonimide groups. The specific synthesis details are shown in Supporting Information. As seen from the optical photographs in Figure S2 (Supporting Information), the prepared membranes are transparent and homogeneous. Figure S3 (Supporting Information) shows the Fourier Transform Infrared (FT‐IR) spectrum of the PFSF, PFS‐NH_2_, PFSI‐PA and PFTSI. The disappearance of the characteristic peak corresponding to sulfonyl fluoride and the appearance of the peak corresponding to the S─N bond indicates that the ‐SO_2_F group is completely converted into a sulfonamide group. The prominent band in the 3600–3200 cm^−1^ region is associated with the P‐OH stretching of PFSI‐PA. The bands at 1070–1280 cm^−1^ correspond to the ‐CF_2_ group, while the band at 980 cm^−1^ is linked to the C─O─C group. In Figure 1b, when compared to the traditional perfluorosulfonic acid ionomers, perfluoroimide multi‐acid ionomers with dual‐active‐center can offer a higher concentration of protons in localized regions, thereby significantly enhancing the ion exchange capacity (IEC). As illustrated in Figure 1c, the IEC values for PFSI‐PA, PFTSI, and PFSA were found to be 1.45, 1.38, and 0.95 mmol g^−1^, respectively. Even under low humidity conditions, its proton conductivity is 1.8 times greater than that of PFSA. Figure S4a (Supporting Information) shows the peaks at ≈1 ppm in the ^31^P Nuclear Magnetic Resonance Spectroscopy (NMR) spectrum corresponding to the chemical shift of the phosphonate group. From the ^1^H NMR spectrum, the peak observed at 8.1 ppm in the ^1^H NMR spectrum of the PFS‐NH_2_ corresponds to the ‐SO_2_‐NH_2_ group. Furthermore, the ^1^H NMR spectrum of PFSI‐PA revealed distinct peaks at 7.9–7.6 ppm, indicative of the chemical shift of hydrogen atoms in the benzene ring (Figure S4c, Supporting Information). Similar chemical shifts of hydrogen atoms can also be observed in the PFTSI hydrogen spectrum (Figure S4d, Supporting Information). The FT‐IR and NMR results further indicate the successful completion of the reaction. Taking PFSI‐PA as an example, Figure 1e demonstrates that the dual‐activity‐center proton‐exchange membrane serves as the core of the membrane electrode, allowing protons to be transported inside it. The sulfonimide and phosphonate groups synergistically offer multiple proton transfer pathways. One pathway relies on a vehicle mechanism that hinges on hydrated hydrogen ions, while the other involves a proton hopping mechanism between acidic functional groups. The structure of the multi‐acid side chain establishes a unique hydrogen bond network with water molecules, which is advantageous for proton transfer.

### 2.2 Proton Conductivity and Fuell Cell Performance

**Figure 2:**
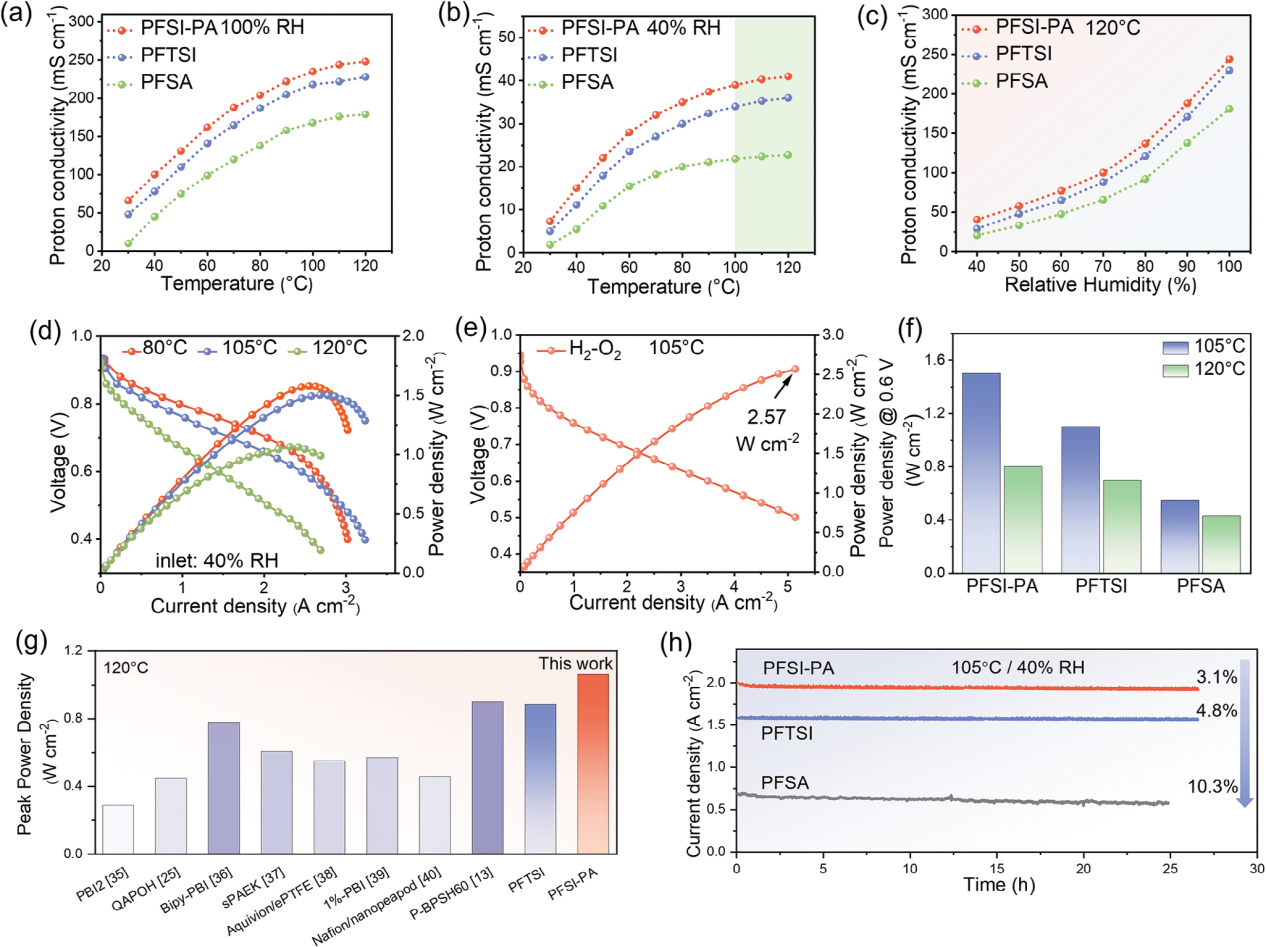
In‐plane proton conductivity of PFSI‐PA, PFTSI and PFSA membranes at different conditions: a) 100% RH, b) 40% RH and c) 120 °C. d) Polarization and power densities curves of PFSI‐PA at 40% RH. e) Polarization and power densities curves of PFSI‐PA at 105 °C/40%RH with H_2_/O_2_. f) Power densities comparison of as‐prepared MEAs under 105 and 120 °C. g) Comparison of peak power density at 120 °C for this work with the recently reported proton exchange membranes. h) Long‐term durability profile at 0.65 V under 105 °C/40% RH.

Proton conductivity quantifies the proton transport capability of a proton exchange membrane. At 100% relative humidity (RH), the proton exchange membrane achieves a highly hydrated state, leading to notably increased conductivity levels for PFSI‐PA and PFTSI at various temperatures compared to PFSA (**Figure** **2**a). Specifically, at 80 °C/100% RH, the conductivities of PFSI‐PA, PFTSI, and PFSA were recorded at 208, 185, and 140 mS cm^−1^, respectively (Figure S5, Supporting Information). Significant differences in conductivity were also observed under low humidity conditions, where PFSI‐PA, PFTSI, and PFSA exhibited conductivities of 38, 28, and 17 mS cm^−1^, respectively, at 40% RH. Furthermore, the conductivity of the prepared membrane increased with rising temperature at 40% RH, although the increment was not substantial (Figure 2b). Remarkably, the conductivity of PFSI‐PA reached 41 mS cm^−1^ at 120 °C, showcasing a 1.8‐time increase compared to that of PFSA (22 mS cm^−1^). This suggests that the perfluoroimide multi‐acid ionomers with dual active centers can sustain excellent proton transport capability even under low humidity conditions and holds promise for application in high‐temperature fuel cells. The conductivity trend with humidity at 120 °C mirrors that observed at 80 °C. Illustrated in Figure 2c, the conductivity of PFSI‐PA peaks at an impressive 244 mS cm^−1^ at 100 °C, surpassing PFTSI at 230 mS cm^−1^ and PFSA at 181 mS cm^−1^. This underscores that membranes featuring a multi‐acid side chain structure can uphold superior proton transport capacity compared to traditional PFSA under varying humidity levels.

To further investigate the potential practical application of the prepared membrane, the performance of membrane electrode assemblies (MEAs) based on PFSI‐PA, PFTSI, and PFSA membranes was evaluated in H_2_/air and H_2_/O_2_ fuel cells. Figure S6a (Supporting Information) displays the polarization curves of the MEAs tested at 80 °C/100% RH. The peak power density of PFSI‐PA reaches 1.63 W cm^−2^, 1.35 W cm^−2^ for PFTSI, and 1.19 W cm^−2^ for PFSA. As the current density increases, the power output gradually rises but starts to decline after reaching the peak power density. This is due to the generation of excess water at high current densities, leading to flooding and impacting mass transfer.^[^ ^34^ ^]^ Lowering the inlet humidity to 40% improves the mass transfer issues of PFSI‐PA at high current density, enabling a peak power density of 1.57 W·cm^−2^ at 80 °C (Figure 2d) and 1.5 W cm^−2^ at 105 °C. At 120 °C and 40% RH, PFSI‐PA demonstrates a power output of 1.06 W cm^−2^. As the temperature rises, the fuel cell's performance gradually deteriorates. This decline may be due to the rapid evaporation of water within the cell, hindering the actual humidity from reaching 40%. In the H_2_/O_2_ fuel cell test (Figure 2e), PFSI‐PA achieves a power output of 2.57 W cm^−2^, with the current density exceeding 5A·cm^−2^ and no occurrence of concentration polarization. As shown in Figure S7 (Supporting Information), under low humidity conditions, all membrane electrodes exhibit a trend of declining performance during temperature escalation, and the High‐Frequency Resistance (HFR) also increases with rising temperatures. Remarkably, ionomers with dual functional groups outperform monofunctional perfluorosulfonic acid polymers significantly under low humidity conditions. The performance of PFSI‐PA at 105 °C exhibits a slight decrease compared to that at 80 °C, whereas PFSA undergoes a sharp decline. As the temperature rises to 120 °C with an inlet humidity of 40%, the power density of PFSA only reaches 0.5 W cm^−2^, while PFSI‐PA achieves a commendable 1.06 W cm^−2^. At 120 °C and 40% RH, the HFR of PFSI‐PA is 87 mΩ cm^2^, that of PFTSI is 103 mΩ cm^2^, and that of PFSA is 121 mΩ cm^2^, demonstrating the exceptional proton transport capability of PFSI‐PA (Figure S7c, Supporting Information). The power output of PFSI‐PA and PFTSI at different temperatures is higher than that of PFSA at 0.6 V (Figure 2f). Figure 2g shows the excellent performance of the PFSI‐PA in terms of peak power density at 120 °C, which exceeds the results reported in other studies, indicating that the performance of the present work at high temperatures is already at the international leading level.^[^ ^13^, ^25^, ^35^, ^36^, ^37^, ^38^, ^39^, ^40^ ^]^

The H_2_ crossover current density is a significant parameter for characterizing membrane integrity. The membrane electrode assembly underwent additional analysis for hydrogen crossover using linear scanning voltammetry. As illustrated in Figure S8 (Supporting Information), all MEAs exhibited a low hydrogen crossover value, measuring <1 mA cm^−2^. This outcome suggests that all the prepared membranes demonstrate good integrity and possess adequate mechanical strength to meet the operational requirements of fuel cells. The Electrochemical Impedance Spectroscopy analyses of the MEAs are based on various membranes at a voltage of 0.65 V, and the impedance values can be obtained by fitting the equivalent circuit diagram model in the inner diagram of Figure S9a (Supporting Information). In impedance analysis, the symbol R_m_ mainly represents the impedance of the membranes, R_t_ represents the charge‐transfer impedance, and R_mt_ represents the mass‐transfer impedance. This is supported by the EIS results, which indicate that the ohmic impedance of PFSI‐PA is merely 0.026 Ω cm^2^ at 80 °C, 0.054 Ω cm^2^ at 105 °C and 0.062 Ω cm^2^ at 120 °C (Figure S9, Supporting Information). The ohmic impedance of PFSI‐PA and PFTSI is consistently lower than that of PFSA under the same conditions.

Figure 2h displays the current density versus time curves at 0.65 V. Operating at 105 °C and 40% RH for 28 h, the current density of PFSI‐PA decreased by 3.1%, PFTSI decreased by 4.8%, while PFSA showed a more pronounced decline of 10.3% after a 25‐hour operation period. This exceptional durability can be attributed to the high‐temperature stability of the PFMA ionomers. The membrane electrode prepared using the proton exchange membrane with dual active centers exhibited superior performance compared to PFSA, particularly PFSI‐PA, highlighting its promising application prospects in future high‐temperature fuel cells. This superiority can be attributed to the microstructure, as well as the physical and chemical properties of the membrane itself. Subsequently, the following discussion will delve into the detailed relationship between structure and performance.

### 2.3 Mechanical and Thermal Properties

**Figure 3:**
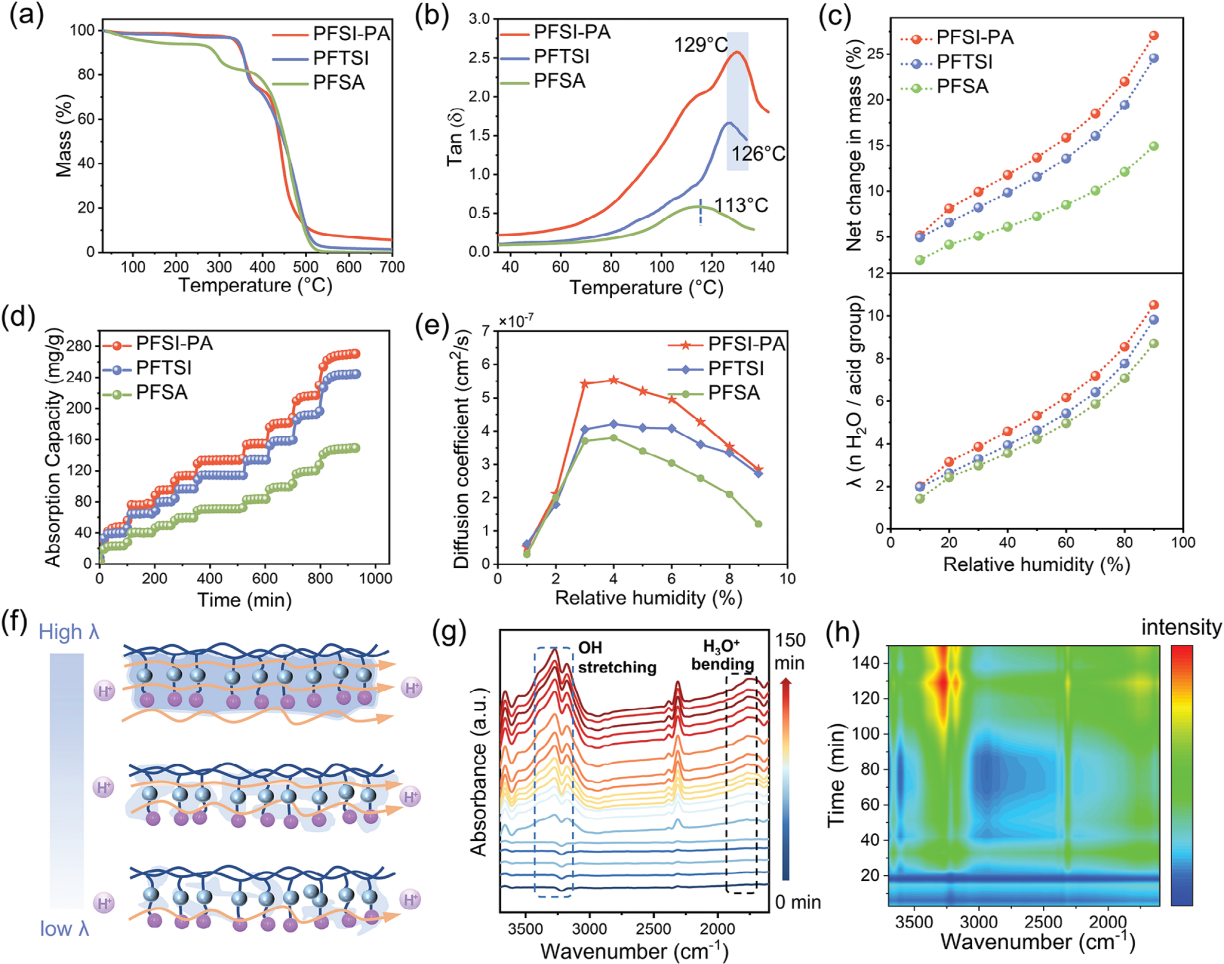
The properties of the PFSI‐PA, PFTSI, and PFSA membranes: a) The TGA curves. b) DMA spectrum. c) Moisture sorption isotherms and λ values at 80 °C. d) Sorption kinetics at 80 °C. e) Water diffusion coefficients at different relative humidity. f) Schematic diagram of the changes in the conduction path of protons at different hydration levels. g) The in suit‐FTIR test of PFSI‐PA. h) the 2D contour map of in‐situ infrared testing.

To evaluate the thermal stability of the PEMs, thermogravimetric analysis and Dynamic Mechanical Analysis (DMA) were conducted. In **Figure** **3**a, it is apparent that all fabricated membranes display negligible thermal weight loss until reaching 330 °C. The tanδ in DMA is related to the α relaxation, which is commonly used to determine the glass transition temperature (*Tg*) for membranes. The PFSI‐PA shows a *Tα* of 129 °C, PFTSI is 126 °C, and PFSA is 113 °C, respectively (Figure 3b). This phenomenon may be attributed to the enhanced electrostatic interactions present within the ionic aggregates of PFMA, serving as crosslinks that impede the long‐range motion of the chain segments. Moreover, the rigid benzene rings within the side chains introduce steric hindrance, thereby diminishing the flexibility of the side chains.^[^ ^41^, ^42^ ^]^ Figure S10 (Supporting Information) depicts the stress versus strain curves of the PEMs. In comparison to PFSA, the PFSI‐PA and PFTSI membranes exhibit exceptional breaking strength (exceeding 20 MPa) and elongation (exceeding 100%). This finding elucidates why PFSA‐based fuel cells exhibit poor performance above 105 °C, whereas PFSI‐PA and PFTSI demonstrate robust performance at elevated temperatures and exhibit excellent long‐term durability.

### 2.4 Water Transport Behavior

The water transport behavior plays a pivotal role in proton transportation.^[^ ^43^ ^]^ Ensuring the proper wetting state of the membrane through gas humidification is essential for effective fuel cell operation. Dynamic Vapor Sorption (DVS) was utilized to investigate the water diffusion behavior of the samples at 80 °C. Figure 3d depicts the water vapor sorption isotherms for the PFSI‐PA, PFTSI, and PFSA samples. As humidity increases, the net mass of the samples gradually rises. Particularly noteworthy is the substantial mass increase observed for PFSI‐PA, reaching 270 mg g^−1^ at 90% RH. The water diffusion coefficients at various humidity levels were calculated using Equation (2).^[^ ^44^ ^]^ The diffusion coefficients of the three samples displayed a consistent trend of initial increase followed by a decrease, likely attributable to the reduction in free volume within the membrane. Notably, the maximum diffusion coefficients for the PFSI‐PA, PFTSI, and PFSA samples were recorded at 40% humidity, measuring 5.53 × 10^−7^ cm^2^ s^−1^, 4.21 × 10^−7^ cm^2^ s^−1^, and 3.82 × 10^−7^ cm^2^ s^−1^, respectively (Figure 3e). Samples with dual active sites exhibited significantly elevated water diffusion coefficients across all humidity levels. The parameter λ is employed to characterize the relationship between water content and the number of protogenic acid groups, critical for water transport within the membrane. The λ value, obtained from Equation (3), demonstrates the exceptional water absorption capacity of PFSI‐PA, with a hydration number of 10.5 at 90% RH, while for PFTSI and PFSA, the values are 9.8 and 8.7 respectively under the same conditions (Figure 3c). This outcome can be attributed to the strong water uptake capability, where PFSI‐PA and PFTSI can achieve 27% and 24.5% at 90% RH, respectively. It is noteworthy that the water absorption capacity, as indicated by the λ value, of PFMA surpasses that of PFSA even under low humidity conditions. This improved water absorption performance can be ascribed to the plentiful acidic active sites present in these polymers, fostering the establishment of multiple hydrogen bonds with water molecules. Elevated humidity levels signify increased hydration, leading to the creation of additional transmission pathways that expedite water and proton transport, aligning with the findings of previous proton conductivity tests (Figure 3f).

In‐situ infrared spectroscopy was employed to investigate the dynamic changes in the microstructure of PFSI‐PA during water absorption (Figure 3g,h). Infrared spectra were captured both before and during illumination, utilizing a continuous scanning mode to ensure real‐time monitoring of the water absorption process. The increasing intensity peak within the wave velocity range of 3400–3100 cm^−1^ corresponds to the hydroxyl peak of water, while the intensifying peak within the range of 1860–1650 cm^−1^ correlates to the hydronium ion. These observations suggest that during the hydration process of PFSI‐PA, protons were ionized and bonded with water molecules to form hydrated protons.

To ascertain the maximum water absorption capacity of the membranes, they were fully immersed in 80 °C water for 12 h. In contrast to the single ion structure, the polymer featuring dual acidic groups exhibited significant water absorption and volume expansion. From Figure S11 (Supporting Information), the water uptake (WU) of the PFSI‐PA membrane, measured at 32.5 wt.%, exceeded that of the other membranes, showcasing a substantial degree of dimensional swelling (14.8%). Conversely, PFSA displayed a lower WU of 18.8% and reduced swelling (8.3%). This finding suggests that membranes with side chains containing multiple acid functional groups exhibit enhanced water absorption, with longer side chains contributing to increased free volume, allowing for the accommodation of a larger number of water molecules.

### 2.5 Microscopic Morphology and Structural Analysis

Figure S12 (Supporting Information) presents their corresponding scanning electron microscope (SEM) cross‐sectional images, demonstrating a uniform thickness of ≈16 µm. In Figure S13a,b (Supporting Information), transmission electron microscopy (TEM) images of PFSI‐PA and PFTSI are depicted. The dark regions depicted in these images signify dense and uniformly distributed hydrophilic clusters, while the light‐colored areas correspond to the backbone regions linked with the tetrafluoroethylene main chain. The phase‐separation microstructures of the prepared PEMs were further analyzed using Atomic Force Microscopy (AFM). Figure S13c,d (Supporting Information) display clear AFM images where the dark regions correspond to hydrophilic areas, while the bright areas indicate the hydrophobic region containing the tetrafluoroethylene skeleton. It is noteworthy that the PFSI‐PA and PFTSI membranes display distinct microphase separation structures with evident connectivity in the hydrophilic regions. These observations suggest that the inclusion of hydrophilic acid groups facilitates the segregation of hydrophobic and hydrophilic phases, thereby fostering the development of proton transport channels.

**Figure 4:**
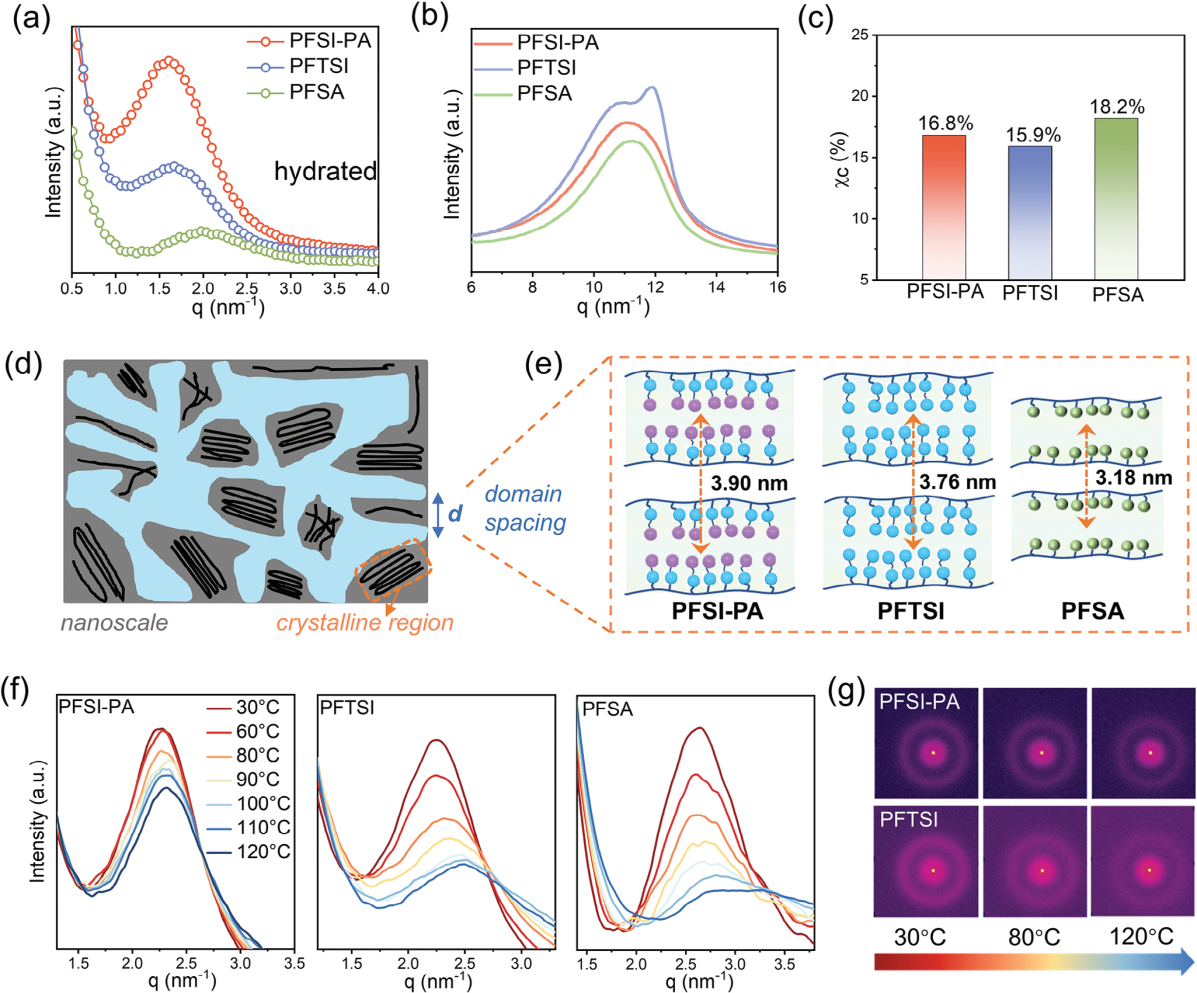
a) The SAXS profiles of the PFSI‐PA, PFTSI and PFSA membranes at hydrated station. b) The WAXS profiles of the PFSI‐PA, PFTSI and PFSA membranes. c) The crystallinity of the PFSI‐PA, PFTSI and PFSA membranes. d) Schematic diagram of the hydrophilic channels (blue) and crystalline regions (dark) of the membrane at the nanoscale. e) The domain spacing between the hydrophilic channels of the prepared membrane in the hydrated state. f) The SAXS profiles of the PFSI‐PA, PFTSI and PFSA membranes at different temperatures. g) 2D SAXS scattering images of the PFSI‐PA and PFTSI membranes.

Moreover, Small angle X‐ray scattering (SAXS) was employed to examine the microscopic morphology of dry and hydrated membranes. The hydrophilic side chains and hydrophobic main chains form a microphase separation structure. **Figure** **4**d illustrates the intertwining of the crystalline region and the water channel region. The ionomer peak, commonly extracted from the scattering data, corresponds to a structural correlation length for hydrophilic domains, interpreted as the spacing between water domains on the nanometer scale.^[^ ^45^ ^]^ The domain spacing (d‐spacing) value was calculated using Equation (6). In Figure 4a, all hydrated samples display a distinct ionomer peak associated with hydrophilic clusters.^[^ ^46^ ^]^ The ionomer peak (ionic domain) is detected within the range of 1.5–2.5 nm^−^¹, with specific q values of 1.61 nm^−^¹ for PFSI‐PA, 1.67 nm^−^¹ for PFTSI, and 1.97 nm^−^¹ for PFSA. The d‐spacing values for PFSI‐PA are determined to be 3.90 nm, 3.76 nm for PFTSI, and 3.18 nm for PFSA, as shown in the Figure 4e. This result demonstrates that the membrane incorporating dual acidic functional groups forms a high‐density local ion domain, creating a larger proton transport channel, enhancing regional connectivity, and facilitating the rapid transport of protons. Furthermore, this outcome supports the previous conductivity and fuel cell test results.

Another peak is identified at slightly higher q values (8–14 nm^−^¹), corresponding to the crystalline wide‐angle X‐ray scattering (WAXS) peak of the polytetrafluoroethylene main chain (Figure 4b). PFSA demonstrates the highest crystallinity at *χc* of 18.2%, while the crystallinity of the other three samples experiences a minor decrease (Figure 4c). This indicates that the side chain modification grafting strategy can increase the number of acid sites without significantly reducing crystallinity, as the ‐CF_2_ unit of the main chain remains unchanged. And the High crystallinity ensures thermal stability.

SAXS test conducted with a gradual temperature increase ranging from 30 to 120 °C for dry membranes. In Figure 4f, the q value and peak intensity of PFSI‐PA exhibit minimal variation, remaining ≈2.48 nm^−1^. In contrast, for PFTSI, the q value transitions from 2.23 to 2.46 nm^−1^. Additionally, the q value of PFSA as the temperature increases, and the intensity of the peak at 120 °C diminishes significantly, indicating the disruption of its nanocluster structure. The 2D SAXS scattering images of PFSI‐PA and PFTSI are illustrated in Figure 4g, showing distinct scattering rings that correspond to the periodic distance d associated with the phase separation behavior within the membrane. The q values of PFSI‐PA and PFTSI remain relatively constant across temperature variations, with minor fluctuations, demonstrating their capacity to maintain a stable phase‐separation structure even at elevated temperatures. This is one of the reasons why PFSA‐based fuel cells exhibit extremely poor high‐temperature performance.

### 2.6 Density Functional Theory Calculations and Molecular Dynamics Simulations

**Figure 5:**
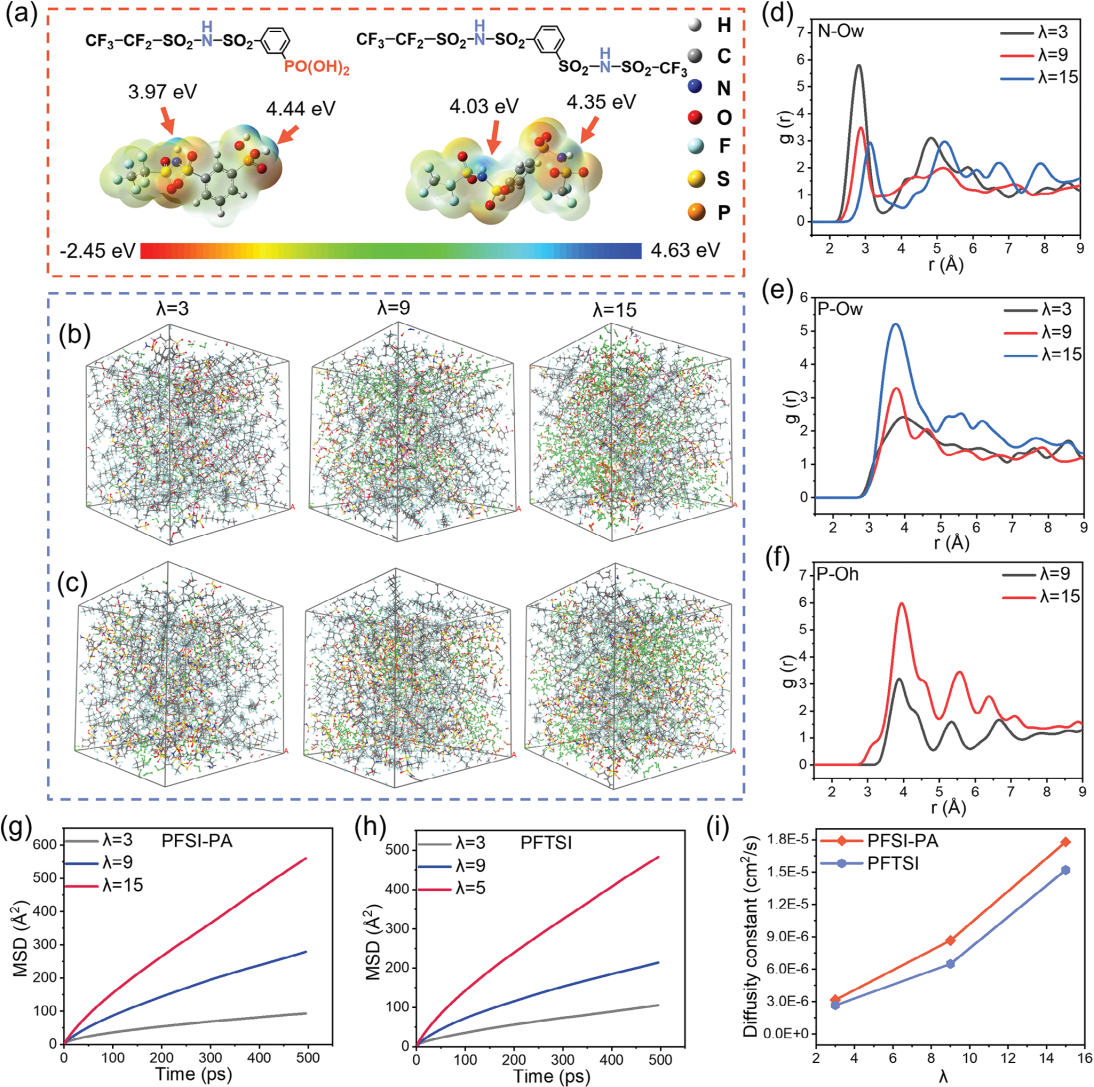
a) Electrostatic potential (ESP) of PFSI‐PA and PFTSI, respectively. Simulated snapshots of b) PFSI‐PA and c) PFTSI at different hydration levels. Orange‐hydronium ions, Green‐water molecules. PFSI‐PA RDFs at different hydration levels for d) N‐Ow, e) P‐Ow and f) P‐Oh; N‐Nitrogen in sulfonyl imide group, phosphorus in phosphonic acid group, Ow‐Oxygen in water molecule, Oh‐Oxygen in hydronium ion. MSDs for water molecules at different hydration levels of g) PFSI‐PA and h) PFTSI. i) Diffusion coefficients of water molecules for PFSI‐PA and PFTSI from MSDs.

To delve deeper into the distinctions in the proton dissociation capabilities among the two distinct multi‐acid side chain structures, DFT simulations were employed.^[^ ^47^, ^48^ ^]^ The two structural configurations illustrated in **Figure** **5**a were utilized to substitute the side chains of PFSI‐PA and PFTSI, respectively. Subsequently, the electrostatic potential (ESP) distribution of their lowest energy structures was computed, where red and blue regions denote electron‐rich and electron‐poor areas, respectively. Elevated electrostatic potential surrounding a hydrogen atom signifies a higher electron deficiency, making the hydrogen atom more predisposed to dissociation. The two acidic functional groups within the side chain exhibit notable electrostatic potential values, indicating their proclivity for ionization. The initial acidic site in PFSI‐PA and PFTSI is sulfonimide, with respective ESP values of 3.97 and 4.03 eV, highlighting variability in ionization energy even when the proton occupies the same position due to the overall side chain influence. Furthermore, the ESP value at phosphonic acid groups (4.44 eV) of PFSI‐PA, surpassing the sulfonimide (4.35 eV) of PFTSI. This indicates that the hydrogen atoms at the phosphonic acid sites are more prone to lose electrons and dissociate, resulting in stronger acidic characteristics. Which is also the fundamental reason why the PFSI‐PA exhibits higher proton conductivity than the PFTSI.

The durability of the PEM is influenced by chemical degradation. By calculating the energy of the lowest unoccupied molecular orbital (LUMO), the highest occupied molecular orbital (HOMO), and their energy gaps (ΔE), Determine their theoretical ability to resist free radical attack. According to DFT calculation of the electron density distribution of the frontier orbitals of the side chain model, the calculation results are shown in Figure S14a (Supporting Information). The energy gap between LUMO and HUMO of PFSI‐PA is larger, which can better protect the side chain from the attack of free radicals, which may be due to the relatively stable benzene ring structure. We then further verified the above theoretical calculations through Fenton reagent accelerated durability tests, which simulates the attack of HO· and HOO· radicals generated during the actual operation. All membranes appeared to have varying degrees of degradation after 50 h of Fenton's test. The weight remaining curves are presented in Figure S14b (Supporting Information). The PFSI‐PA has superior antioxidative stability (Weight reduction of only 15%) than PFTSI.

Classical molecular dynamics simulations have been employed to investigate the correlation between membrane morphology and the diffusion of hydronium ions (protonated water molecules) and water molecules.^[^ ^49^ ^]^ In this section, we analyze the diverse dynamic characteristics of PFSI‐PA and PFTSI membranes and delineate their variances across different hydration levels. The distribution morphology of water molecules and hydrated protons in the ionic nanophase of PFSI‐PA and PFTSI was investigated. The molecular structure and schematic illustration of the coarse‐grained model are depicted in Figure S15 (Supporting Information), with simulated snapshots shown in Figures 5b,c. Both samples exhibit typical microphase‐separated morphologies, where the backbone components create a hydrophobic domain, while the perfluoroimide multi‐acid side chain, water molecules, and hydrated protons form a hydrophilic domain. These two domains interlace with each other, forming a bicontinuous structure. To further explore the morphology of these hydrophilic domains, simulated snapshots of each are extracted from the entire system and presented in Figure S16 (Supporting Information). When λ is 3, the sulfonimide and phosphonic acid groups are unable to dissociate protons, causing water molecules and acidic functional groups to attract each other. At λ = 9, both samples exhibit distinctive hydrophilic‐hydrophobic phase separation structures and form prominent hydrophilic channels. When λ is 15, all protons bind with water molecules, resulting in more continuous hydrophilic channels and higher concentrations of hydronium ions. PFSI‐PA develops a larger hydration area, with the channel formed being larger than that in PFTSI. In both samples, the high density of hydrated protons at the channel's core facilitates proton transport along the channel, thereby enhancing proton transport efficiency. This observation is consistent with the findings derived from small‐angle scattering and AFM analyses.

The local structures and interactions within the membranes were analyzed using the radial distribution function (RDF, g(r)) of specific atomic pairs. The sulfonimide group and the phosphonic acid group make the side chains hydrophilic, showing strong interactions with water molecules and hydrated hydrogen ions. Therefore, investigating the interactions of N and P with oxygen atoms of water molecules (Ow) and oxygen atoms of hydrated hydrogen ions (Oh) provides valuable insights. Figure 5d illustrates the RDF of N‐Ow for PFSI‐PA. At λ = 3, the N‐Ow RDF displays its initial peak at 2.8 Å, whereas at a higher hydration level (λ = 15), the first peak of the N‐Ow RDF shifts to 3.15 Å, suggesting the existence of the first hydration shell around the sulfonimide group. At low humidity, the proximity between the sulfonyl imide group of PFTSI and the water molecule is very close, ≈2.8 Å. As λ increases to 9 and 15, this distance expands due to the ionization of protons, forming hydrated protons in association with water molecules (Figures S17a and S18b, Supporting Information). With increasing hydration levels, the sulfonimide group ionizes all protons, resulting in a net negative charge that attracts positively charged hydrated protons, leading to a separation from water molecules.

The position of the first peak in the N‐Oh RDF at λ = 15 for both PFSI‐PA and PFTSI is 3.3 Å (Figure S17c, Supporting Information). Likewise, the first peak in the RDF of N‐Ow for PFTSI occurs at 3.6 Å for λ = 9 and λ = 15, as depicted schematically in Figure S17a (Supporting Information). In contrast, P‐Ow exhibits a distinct pattern, showing the initial peak at 4 Å at λ = 3, which marginally decreases to 3.7 Å at λ = 9 and 15 (Figure 5e). This suggests that the spatial separations between the sulfonimide and phosphonic acid groups and the water molecules in the primary hydration shell are 2.8 and 3.7 Å, respectively (Figure S18a, Supporting Information). The peak position of the P‐Oh RDF remains approximately constant at ≈4 Å under different humidifies, indicating that the distances between phosphonate and hydrated protons remain consistent regardless of humidity levels (Figure 5f). Figure S17d (Supporting Information) show that the peak position of the Ow‐Oh RDF is ≈2.9 Å, with the distances between water molecules and hydrated hydrogen ions being unaffected by the specific acidic functional group.

The mean‐square displacements (MSD) values were computed as averages for all water molecule atoms (Figure 5g,h) and separately for all hydronium ions (Figure S19, Supporting Information). The MSD values for PFSI‐PA are higher than those for PFTSI, for both hydronium ions and water molecules. The MSD for water molecules and hydronium ions continues to increase with rising hydration levels due to the formation of larger water phase clusters as λ increases. The plots of MSD versus time exhibit a linear trend, indicating a diffusive regime. Notably, the MSD values for hydronium ions are considerably lower than those for water molecules, primarily because hydronium ions, being positively charged, are strongly drawn to the negatively charged protogenic groups, restricting their mobility. At λ = 3, the MSD plot appears almost linear since no hydronium ions are formed at this stage. Figure 5i displays the simulated water diffusion coefficients at various hydration levels, derived from the MSD plots of the current simulations. Across all λ values, the water diffusion coefficients for PFSI‐PA surpass those of PFTSI. Specifically, at λ = 15, the water diffusion coefficient for PFSI‐PA is 1.78 × 10^−5^ cm^2^ s^−1^, while for PFTSI, it is 1.52 × 10^−5^ cm^2^ s^−1^. indicating a larger proton conductive channel in PFSI‐PA. The results from DFT and MD simulations confirm that PFSI‐PA exhibits superior proton dissociation capabilities, tends to develop larger hydrophilic channels at elevated hydration levels, and demonstrates a faster water diffusion rate. These findings offer theoretical backing for the electrochemical tests results.

## 3 Conclusions

In this study, we synthesized Perfluoroimide multi‐acid side (PFMA) ionomers containing sulfonimide and phosphonic acid groups in the side chains. PFMA reduces the IEC while upholding backbone crystallinity, resulting in significantly enhanced water absorption and water transport due to the multiple protogenic acid groups. Additionally, the hydrophobic main chain and hydrophilic side chain architecture establish a distinctive micro‐phase separation structure, creating a pathway for proton transmission and enhancing proton conductivity. The synergistic effect of the sulfonyl imide group and the phosphonic acid group leads to the formation of a larger hydrophilic channel. Simulation calculations also reveal that PFSI‐PA establishes a more extensive hydrophilic area at high hydration levels, thereby enhancing its ability to effectively transport water molecules and hydrated protons. Furthermore, the incorporation of a rigid benzene ring fragment was implemented to diminish side chain flexibility. And the electrostatic interactions within the ionic aggregates impede the long‐range motion of the chain segments, thereby enhancing thermal stability. The synthesized PFSI‐PA membrane demonstrates a conductivity of 244 mS cm^−1^ at 100% RH and 41 mS cm^−1^ at 40% RH, representing a 1.8 times enhancement over that of PFSA. In fuel cell testing, the membrane electrode composed of PFSI‐PA exhibits commendable performance, particularly in low humidity conditions, achieving a notable power output of 1.58 W cm^−2^ under hydrogen‐air conditions at 105 °C and a density of 2.57 W cm^−2^ under hydrogen and oxygen conditions. Furthermore, it exhibits outstanding long‐term durability under operational conditions of 105 °C and 40% RH, with the current density decreasing by only 3.1% over 26.5 h of continuous operation, in contrast to the 10.3% reduction observed in PFSA. When compared to perfluorosulfonic acid with a single acidic functional group, the polyacid side chain structural resin featuring dual active centers showcases superior overall performance. This strategic surpasses the operational temperature limitations of conventional proton exchange membranes and provides valuable insights for the advancement of critical membrane materials for high‐temperature fuel cells.

## Conflict of Interest

The authors declare no conflict of interest.

## Supporting information



Supporting Information

## Data Availability

The data that support the findings of this study are available in the supplementary material of this article.

## References

[1] K. Jiao , J. Xuan , Q. Du , Z. M. Bao , B. A. Xie , B. W. Wang , Y. Zhao , L. H. Fan , H. Z. Wang , Z. J. Hou , S. Huo , N. P. Brandon , Y. Yin , M. D. Guiver , Nature 2021, 595, 361. 34262215 10.1038/s41586-021-03482-7

[2] S. Q. Zhao , Y. C. Liao , R. Wang , G. L. Liu , H. N. Zhang , H. L. Tang , Chem. Eng. J. 2023, 472, 144804.

[3] R. Devanathan , Energy Environ. Sci. 2008, 1, 101.

[4] R. Haider , Y. C. Wen , Z. F. Ma , D. P. Wilkinson , L. Zhang , X. X. Yuan , S. Q. Song , J. J. Zhang , Chem. Soc. Rev. 2021, 50, 1138. 33245736 10.1039/d0cs00296h

[5] A. Chandan , M. Hattenberger , A. El‐kharouf , S. Du , A. Dhir , V. Self , B. G. Pollet , A. Ingram , W. Bujalski , J. Power Sources 2013, 231, 264.

[6] M. Casciola , G. Alberti , M. Sganappa , R. Narducci , J. Power Sources 2006, 162, 141.

[7] N. L. Garland , J. P. Kopasz , J. Power Sources 2007, 172, 94.

[8] H. Nguyen , F. Lombeck , C. Schwarz , P. A. Heizmann , M. Adamski , H.‐F. Lee , B. Britton , S. Holdcroft , S. Vierrath , M. Breitwieser , Sustain. Energy Fuels 2021, 5, 3687.

[9] H. Nguyen , C. Klose , L. Metzler , S. Vierrath , M. Breitwieser , Adv. Energy Mater. 2022, 12, 2103559.

[10] H. Y. Tan , S. Q. Zhao , S. E. Ali , S. H. Zheng , A. K. Alanazi , R. Wang , H. N. Zhang , H. M. Abo‐Dief , B. Bin Xu , H. Algadi , H. D. Li , P. Wasnik , Z. H. Guo , H. L. Tang , J. Mater. Sci. Technol. 2023, 166, 155.

[11] A. Kumar , J. Mater. Chem. A 2020, 8, 22632.

[12] S. H. Mirfarsi , A. Kumar , J. Jeong , M. Adamski , S. McDermid , B. Britton , E. Kjeang , Int. J. Hydrogen Energ. 2024, 50, 1507.

[13] C. H. Park , S. Y. Lee , D. S. Hwang , D. W. Shin , D. H. Cho , K. H. Lee , T. W. Kim , T. W. Kim , M. Lee , D. S. Kim , C. M. Doherty , A. W. Thornton , A. J. Hill , M. D. Guiver , Y. M. Lee , Nature 2016, 532, 480. 27121841 10.1038/nature17634

[14] J. Dai , Y. Zhang , G. Wang , Y. Zhuang , Sci. China Mater. 2021, 65, 273.

[15] L. Cao , H. Wu , Y. Cao , C. Fan , R. Zhao , X. He , P. Yang , B. Shi , X. You , Z. Jiang , Adv. Mater 2020, 32, 2005565. 10.1002/adma.202005565 33179394

[16] X. Wang , Y. He , B. Zhao , C. Du , S. Wang , H. Wang , S. Mao , J. Zhao , Y. Wang , C. Xiong , Sep. Purif 2024, 349, 127909.

[17] J. Song , W. Zhao , L. Zhou , H. Meng , H. Wang , P. Guan , M. Li , Y. Zou , W. Feng , M. Zhang , L. Zhu , P. He , F. Liu , Y. Zhang , Adv. Sci 2023, 10, 2303969. 10.1002/advs.202303969 PMC10602569 37653601

[18] P. Guan , Y. Zou , M. Zhang , W. Zhong , J. Xu , J. Lei , H. Ding , W. Feng , F. Liu , Y. Zhang , Sci. Adv. 2023, 9, eadh1386. 37126562 10.1126/sciadv.adh1386 PMC10132749

[19] A. Z. Al Munsur , B.‐H. Goo , Y. Kim , O. J. Kwon , S. Y. Paek , S. Y. Lee , H.‐J. Kim , T.‐H. Kim , ACS Appl. Mater. Interfaces 2021, 13, 28188. 34125524 10.1021/acsami.1c05662

[20] B. Zhang , Y. Cao , S. T. Jiang , Z. Li , G. W. He , H. Wu , J. Membr. Sci. 2016, 518, 243.

[21] M. Schuster , T. Rager , A. Noda , K. D. Kreuer , J. Maier , Fuel Cells 2005, 5, 355.

[22] N. R. Kang , T. H. Pham , H. Nederstedt , P. Jannasch , J. Membr. Sci. 2021, 623, 119074.

[23] S.‐S. Liu , Q.‐Q. Liu , S.‐Z. Huang , C. Zhang , X.‐Y. Dong , S.‐Q. Zang , Coord. Chem. Rev. 2022, 451, 214241.

[24] T. H. Zhu , B. B. Shi , H. Wu , X. D. You , X. Y. Wang , C. Y. Fan , Q. Peng , Z. Y. Jiang , Ind. Eng. Chem. Res. 2021, 60, 6337.

[25] K. S. Lee , J. S. Spendelow , Y. K. Choe , C. Fujimoto , Y. S. Kim , Nat. Energy 2016, 1, 16120.

[26] V. Atanasov , A. S. Lee , E. J. Park , S. Maurya , E. D. Baca , Nat. Mater. 2021, 20, 370. 33288898 10.1038/s41563-020-00841-z

[27] S. Kang , M. J. Park , ACS Macro Lett. 2020, 9, 1527. 35617073 10.1021/acsmacrolett.0c00629

[28] W. Zhang , S. Zhao , R. Wang , A. Zhang , Y. Huang , H. Tang , Adv. Compos. Hybrid Mater 2023, 6, 60.

[29] S. C. Savett , J. R. Atkins , C. R. Sides , J. L. Harris , B. H. Thomas , S. E. Creager , W. T. Pennington , D. D. DesMarteau , J. Electrochem. Soc. 2002, 149, A1527.

[30] L. Assumma , C. Iojoiu , G. A. Ari , L. Cointeaux , J. Y. Sanchez , Int. J. Hydrogen Energ. 2014, 39, 2740.

[31] Y. Liao , S. Zhao , G. Liu , H. Li , J. Shuai , L. Wang , B. Liu , H. Tang , Chem. Eng. J. 2024, 488, 150971.

[32] H. Eskandari , D. K. Paul , A. P. Young , K. Karan , ACS Appl. Mater. Interfaces 2022, 14, 50762. 36342365 10.1021/acsami.2c12667

[33] A. Kusoglu , K. Vezzù , G. A. Hegde , G. Nawn , A. R. Motz , H. N. Sarode , G. M. Haugen , Y. Yang , S. Seifert , M. A. Yandrasits , S. Hamrock , C. M. Maupin , A. Z. Weber , V. Di Noto , A. M. Herring , Chem. Mater 2019, 32, 38.

[34] H. Choi , H. Jang , J. Kim , O. Kwon , H. Yoo , H. Cha , S. Jeong , Y. So , T. Park , J. Power Sources 2023, 580, 233311.

[35] K. Firouz Tadavani , A. Abdolmaleki , M. R. Molavian , S. Borandeh , E. Sorvand , M. Zhiani , Energy Fuels 2017, 31, 11460.

[36] M. R. Berber , N. Nakashima , J. Membr. Sci. 2019, 591, 117354.

[37] H. L. Cheng , J. M. Xu , L. Ma , L. S. Xu , B. J. Liu , Z. Wang , H. X. Zhang , J. Power Sources 2014, 260, 307.

[38] P. Xiao , J. S. Li , H. L. Tang , Z. Wang , M. Pan , J. Membr. Sci. 2013, 442, 65.

[39] J. Peng , X. Fu , J. Luo , Y. Liu , L. Wang , X. Peng , J. Membr. Sci. 2022, 643, 120037.

[40] K. T. Park , J. H. Chun , S. G. Kim , B. H. Chun , S. H. Kim , Int. J. Hydrogen Energ. 2011, 36, 1813.

[41] K. A. Page , K. M. Cable , R. B. Moore , Macromolecules 2005, 38, 6472.

[42] V. Di Noto , M. Piga , G. A. Giffin , K. Vezzù , T. A. Zawodzinski , J.Am.Chem.Soc 2012, 134, 19099. 23102554 10.1021/ja3071336

[43] P. W. Majsztrik , M. B. Satterfield , A. B. Bocarsly , J. B. Benziger , J. Membr. Sci. 2007, 301, 93.

[44] D. J. Burnett , A. R. Garcia , F. Thielmann , J. Power Sources 2006, 160, 426.

[45] A. Kusoglu , A. Z. Weber , Chem. Rev. 2017, 117, 987. 28112903 10.1021/acs.chemrev.6b00159

[46] S. Y. Choi , S. Cho , D. Kim , J. Kim , G. Song , R. Singh , C. Kim , J. Membr. Sci 2021, 620, 118904.

[47] G. Kresse , J. Furthmüller , Phys. Rev. B 1996, 54, 11169. 10.1103/physrevb.54.11169 9984901

[48] S. Grimme , S. Ehrlich , L. Goerigk , J. Comput. Chem. 2011, 32, 1456. 21370243 10.1002/jcc.21759

[49] S. Y. Choi , M. M. Ikhsan , K. S. Jin , D. Henkensmeier , Int. J. Energy Res 2022, 46, 11265.

