# Supplementary material for: Proton Exchange Membrane with Dual‐Active‐Center Surpasses the Conventional Temperature Limitations of Fuel Cells
Source: Adv Sci (Weinh). 2025 Jan 21;12(10):2417259. doi: 10.1002/advs.202417259 (PMC11905064; doi:10.1002/advs.202417259)
Supplement: Supplementary file 1 — Supporting Information [file ADVS-12-2417259-s001.docx]

Proton exchange membrane with dual-active-center surpasses the conventional temperature limitations of fuel cells

Yucong Liao^a&^, Shengqiu Zhao*^b^*^&^, Rui wang^a^, Junjie Zhang^a^, Hao Li^a^, Bingxuan Liu^a^, Yao Li^a^, Aojie Zhang^a^, TianTian^a^, Haolin Tang^a, b, c ^[[1]](#footnote-1)^*^

*^a^ State Key Laboratory of Advanced Technology for Materials Synthesis and Processing, Wuhan University of Technology, Wuhan 430070, China*

*^b^ National energy key laboratory for new hydrogen-ammonia energy technologies, Foshan Xianhu Laboratory, No.1 Yangming Road, Danzao Town, Nanhai District, Foshan 528200, Guangdong, P.R.China.*

*^c^ Hubei Key Laboratory of Fuel Cell, Wuhan 430070, China.*

^&^ *These authors contributed equally to this work*

** Corresponding author E-mail: thln@whut.edu.cn*

Perfluorosulfonyl fluoride (PFSF, EW = 1050) and the associated Perfluorosulfonic acid (PFSA) polymers were obtained from Wuhan Lvdong Hydrogen Energy Technology Co., Ltd. The solvents and reagents, including N,N-Dimethylformamide (DMF), dimethylsulfoxide (DMSO), benzene-1,3-di(sulphonyl chloride), 3-Bromobenzenesulfonyl chloride, Trifluoromethanesulphonamide, and triethylamine (TEA), were supplied by Aladdin Industrial Corporation located in Shanghai, China. Ethyl acetate and triethyl phosphite were sourced from InnoChem Science & Technology Co., Ltd. in Beijing, China. Alcohol, sodium hydroxide (NaOH), hydrochloric acid (HCl), and sulfuric acid (H₂SO₄) were acquired from Sinopharm Chemical Reagent Corporation. Deionized water with a resistivity of 18.25 MΩ cm was used in the experiments, and all chemicals were utilized as received, requiring no further purification.

**2. Fabrication of membranes**

**2.1 Synthesis of** **Perfluoroalkanesulfonylamides (PFS-NH_2_)**

Perfluoroalkanesulfonylamides (PFS-NH_2_) were produced through the reaction of sulfonyl fluoride with liquid ammonia, as shown in **Figure 1**. The reaction of PFSF with liquid ammonia was conducted at temperatures below -78 °C for three days. After this period, the resulting polymer was treated with a 1M H₂SO₄ solution for 24 hours, then washed to neutralize and dried. The yield of PFS-NH_2_ is about 95%.

**2.2 Synthesis of** **Perfluorosulfonimide phosphonic acid (PFSI-PA)**

Initially, 2 g of PFS-NH_2_ was dissolved in 100 ml of DMF. Then, 1 g of 3-Bromobenzenesulfonyl chloride and 10 ml of TEA were added to this solution, which was stirred at 0°C for three days under a nitrogen atmosphere (see **Figure S1a**). The reaction mixture was extracted with ethyl acetate and subsequently washed with water and alcohol. Following this, the solids were dissolved in DMSO, and 1 g of Triethyl phosphite was incorporated for a reaction period of 24 hours. The resulting solids were then placed in a 3 mol L⁻¹ hydrochloric acid solution for another 24 hours, after which they were washed and dried to obtain the final product, PFSI-PA. The yield of PFS-PA is about 90%.

**2.3 Synthesis of** **Perfluorodisulfonimide (PFTSI)**

A total of 2 g of PFS-NH_2_ was dissolved in 100 ml of DMF. After this, 1 g of benzene-1,3-di(sulphonyl chloride) and 10 ml of TEA were added, and the mixture was stirred at 0°C for three days in a nitrogen atmosphere (see **Figure S1b**). Next, 1 g of Trifluoromethanesulphonamide was added, and the reaction continued for 24 hours. The mixture was then extracted with ethyl acetate and washed with water and alcohol. The resulting solids were immersed in a 1M sodium hydroxide solution for 24 hours, treated with 3 mol L⁻¹ hydrochloric acid, and finally washed and dried to yield the product PFTSI. The yield of PFTSI is about 92%.

**2.4 Membranes preparation**

The membranes PFSI-PA, PFTSI, and PFSA were prepared by our group utilizing the solution casting technique. For instance, a 5 wt% solution of PFSI-PA was created by stirring it in DMSO at 160°C for six hours. This solution was then poured into a glass petri dish and dried at 80°C for 12 hours. The resulting film was annealed at 150°C for two hours to improve crystallinity and remove residual solvent. The prepared membrane underwent a standard treatment, being placed in a 1 M H₂SO₄ solution at 80°C for 12 hours, followed by another 12 hours in deionized water at the same temperature. Finally, the membrane was dried to ensure all moisture was removed. PFTSI and PFSA membranes were prepared using the same procedure as outlined above.

**3 Material Characterization**

**3.1 Instrumentation**

The chemical composition of the synthesized sample was determined using Fourier Transform Infrared spectroscopy (FT-IR) with a Thermofisher Scientific Nicolet6700 instrument. Furthermore, ¹H and ³¹P Nuclear Magnetic Resonance (NMR) spectra were obtained using a Varian 400 MHz NMR spectrometer, with DMSO-d6 as the solvent and tetramethylsilane (TMS) as the internal standard. The morphology of all Polymer Electrolyte Membranes (PEMs) was analyzed through field emission scanning electron microscopy (FE-SEM, Zeiss Ultra Plus). Additionally, transmission electron microscopy (TEM, IEM-2100F) was used to assess morphology, chemical composition, and elemental distribution.

Simultaneous thermogravimetric analysis (TGA) was performed with a TGA 1 star® system (STA449F3) to evaluate the thermal properties of the samples, heating them to 800°C at a rate of 10°C/min under nitrogen flow. The mechanical properties of the membranes were tested at ambient temperature using a universal testing machine (Instron 3343) with 10 mm × 80 mm samples, applying a strain rate of 10 mm/min with a 500 N load cell. The thermomechanical properties were analyzed in a temperature range of 30-160°C using the PerkinElmer DMA8000 Dynamic Thermomechanical Analyzer. Surface morphology was examined using a Seiko SPA400 atomic force microscope (AFM) in tapping mode, with the membrane being hydrated for one hour at 100% relative humidity prior to imaging. Lastly, the water diffusion behavior of the membrane at varying relative humidity levels was assessed using a Dynamic Vapor Sorption (DVS) Analyzer from Beishide Instrument Technology in Beijing.

**3.2 Ion exchange capacity (IEC)**

The ion exchange capacity (IEC), which indicates the amount of exchangeable ions in milliequivalents, was assessed through acid-base titration methods. To carry out these experiments, all membrane samples were thoroughly dried and weighed immediately. The membrane samples were then shredded and soaked in a saturated NaCl solution, stirring for 24 hours to ensure proper replacement of H⁺ ions. For the titration, a 0.1 M NaOH solution was employed, using an automatic titrator (Metrohm, 916 Ti-Touch) that was calibrated with a standard buffer solution prior to the procedure. The IEC was determined using equation (1):

$\mathrm{IEC}(mmol g^{-1})=\frac{V_{\mathrm{NaOH}}\times C_{\mathrm{NaOH}}}{m_{dry membrane}}$ (1)

In this equation, *V*_NaOH_ and *C*_NaOH_ represent the volume and concentration of the titrating solution used, respectively, and m*_dry_* _membrane_ weights are recorded in advance after the membrane sample has been dried.

**3.3 Water diffusion coefficient**

The diffusion constants for thin films were calculated using the diffusion equation originally proposed by Crank and Park. To find the diffusion constant, film samples were placed in a Dynamic Vapor Sorption (DVS) system and subjected to moisture-saturated nitrogen at 80°C. The weight change was monitored as the relative humidity increased from 10% to 90%, adhering to standard adsorption equilibrium conditions of 0.1 mg per 60 minutes. The initial kinetics of adsorption into bodily fluids can be described by the following equation.

$\frac{M_{t}}{M_{\infty}}=\frac{4}{d}\sqrt{\frac{Dt}{\pi}}$ (2)

In this equation, M_t_ represents the amount adsorbed at time t, M∞ represents the amount adsorbed at thermodynamic equilibrium, and D represents the diffusion constant.

**3.4 Hydration level (λ)**

The hydration level (λ) is defined as the ratio of water molecules to protogenic acid groups in PFSI-PA, PFTSI, and PFSA. Here, Δm represents the weight difference between the wet and dry membranes, m is the weight of the dry membrane, EW denotes the equivalent weight, and M_H₂O_ is the molar mass of water.

$\lambda=\frac{\Delta m}{m}*\frac{EW}{M_{H_{2}O}}$ (3)

**3.5 Water uptake and dimensional swelling**

The water uptake (WU) and dimensional swelling (DS) of the PEMs were measured following a specific procedure. First, the PEMs were placed in a vacuum oven at 100°C and dried for 8 hours. The weight (m_dry_) and length (L_dry_) of the dried PEMs were then measured and noted. Subsequently, the dried PEMs were soaked in deionized water at 80°C for 12 hours. After soaking, excess water on the surface of the membranes was carefully removed, and the weight (m_wet_) and length (L_wet_) of the wetted PEMs were recorded. The WU and DS values were calculated using equations (4) and (5), respectively.

$\mathrm{WU}\left( \% \right)=\frac{m_{wet}-m_{dry}}{m_{dry}}$ (4)

$\mathrm{DS}\left( \% \right)=\frac{L_{wet}-L_{dry}}{L_{dry}}$ (5)

We assessed its hydrated state by subjecting the membrane to complete water absorption through soaking.

**3.6** **Small- and Wide- angle X-ray scattering (SAXS and WAXS) analysis**

The membranes' microstructure was analyzed through small- and wide-angle X-ray scattering (SAXS and WAXS) experiments conducted with the Xenocs Xeuss3.0 system. This setup measured the scattered wave vector (q) and the corresponding intensity curve. Subsequently, the d-spacing value was determined using Bragg's law:

$d=\frac{2\pi}{q}$ (6)

**3.7 Proton conductivity**

The in-plane proton conductivity of PEMs was examined using a conductivity measurement system that included a Membrane Test System (MTS740, Scribner). Impedance values for the membranes were obtained through alternating current electrochemical impedance spectroscopy (EIS) across various temperatures and relative humidity levels. The conductivity of the PEMs was determined using the following equation:

$\sigma= \frac{L}{R\times S}$ (7)

Where *L* (cm), *S* (cm), and *R* (Ω) are the length between the collectors, the cross-sectional area, and the resistance obtained from the EIS, respectively.

**3.8 Ex-situ Chemical Durability**

The PEMs were exposed to Fenton's reagent (30 wt% H₂O₂ and 20 ppm FeSO₄⋅7H₂O) at 80°C for 50 hours to evaluate their chemical durability ex situ. To maintain the reactivity of the Fenton reagent, the solution was refreshed every hour, and the remaining weight of the PEMs was recorded.

**3.9 Theoretical calculations**

All quantum chemical calculations were performed using the GAUSSIAN-16 software package. The structures studied were fully optimized with the B3LYP-D3(BJ) functional and the 6-311G(d,p) basis set, utilizing the implicit SMD solvent model. Vibrational frequencies were calculated at the same theoretical level to analyze the characteristics of stationary points. The Electrostatic Potential (ESP) was further examined using MultiWFN 3.8. All computations were conducted with the Vienna Ab-initio Simulation Package (VASP) based on density functional theory (DFT). Electron exchange-correlation interactions were described using the generalized gradient approximation (GGA) with the PBE functional, and the projector augmented wave (PAW) method was employed for the electron-ion interactions. Additionally, the DFT-D3 method was applied to account for long-range van der Waals forces. Integration in the irreducible Brillouin zone used the Monkhorst-Pack scheme, with a kinetic energy cutoff of 450 eV for plane wave expansion. Both lattice parameters and ionic positions were fully relaxed, achieving total energy convergence within 10⁻⁵ eV per formula unit, and final forces on all ions remained below 0.02/Å. Molecular dynamics simulations were executed using the Forcite module in Materials Studio. Initial molecular structures were based on DFT calculation outcomes, including deprotonated forms corresponding to various hydration levels. Final simulation structures were created from Hydronium, water, and the deprotonated structures assembled via the Amorphous Cell module. The COMPASSIII force field, known for accurately modeling hydrogen-bond networks and proton transfer in electrolyte systems, was used for these simulations. The topological structure was optimized using the conjugate gradient method to remove atomic overlaps and unreasonable configurations. A 1 ns dynamic relaxation at 353 K under the NPT ensemble with the Nose thermostat was conducted to adjust the volume to atmospheric pressure density, followed by a 1 ns NVT relaxation to reach an equilibrium state. Throughout the molecular dynamics process, the diffusion rate of hydrated protons and the radial distribution function (RDF) between components were analyzed, with electrostatic potential summations performed using the PPPM method.

**3.10 Fabrication of membrane electrode assembly (MEA) and fuel cell tests**

The preparation of the MEA and its catalyst layers adhered to established protocols. In brief, a mixture consisting of catalyst (Pt/C = 50%), Nafion solution, isopropyl alcohol, and deionized water was created in specific ratios. This catalyst ink was made homogeneous through 30 minutes of ultrasonic treatment. Following this, the catalyst was evenly applied to PTFE substrates using a painting technique. These layers were then hot-pressed onto both sides of the PEM at 150°C and 900 psi for 150 seconds. Afterward, the PTFE substrate was removed, yielding a catalyst-coated membrane (CCM) with catalyst loadings of 0.1 mg/cm² for the anode and 0.4 mg/cm² for the cathode. To finalize the MEA assembly, gas diffusion layers (GDL) of 200 μm thickness were affixed to both sides of the CCM. The completed MEA was placed within a single-cell testing fixture, featuring a serpentine flow field plate and a fuel cell clamp.

The performance of the single cell was assessed using a fuel cell testing system (HTS-125s, Hephas Energy Company) equipped with constant potential measurement tools (Module 885, Scribner Associates Inc.). Hydrogen and air were fed to the anode and cathode at stoichiometric ratios of 1.5 for hydrogen and 2.5 for air, with minimum flow rates of 1 L/min for hydrogen and 2 L/min for air. During the fuel cell test, the back pressure was set to 150 Kpa. For another set of tests, hydrogen and oxygen were supplied at stoichiometric ratios of 1.5 each. To evaluate hydrogen crossover in the PEMs, linear scanning voltammetry (LSV) was performed at 25°C without back pressure, with a scan rate of 2 mV/s from 0 to 0.75 V. The current density at 0.4 V was identified as the hydrogen crossover value for the PEMs. During the LSV measurements, fully humidified hydrogen (0.2 L/min) and nitrogen (0.2 L/min) were supplied to the anode and cathode of the MEA, respectively.


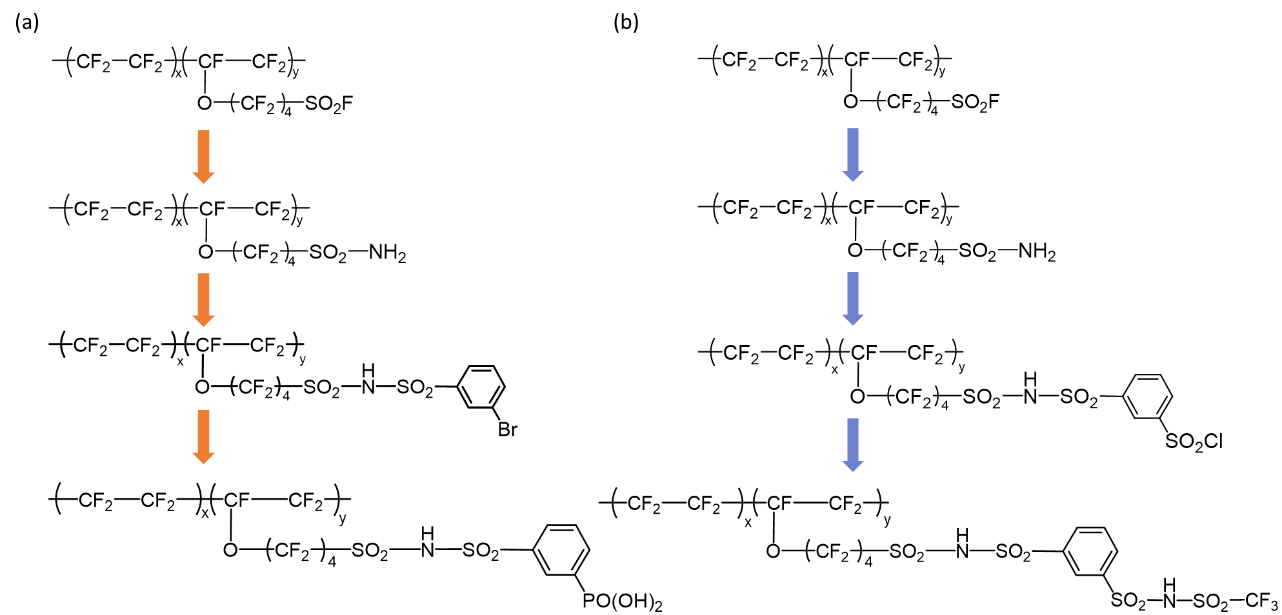


**Figure S1**. The schematic diagram of the synthesis process of (a) PFSI-PA and (b) PFTSI.


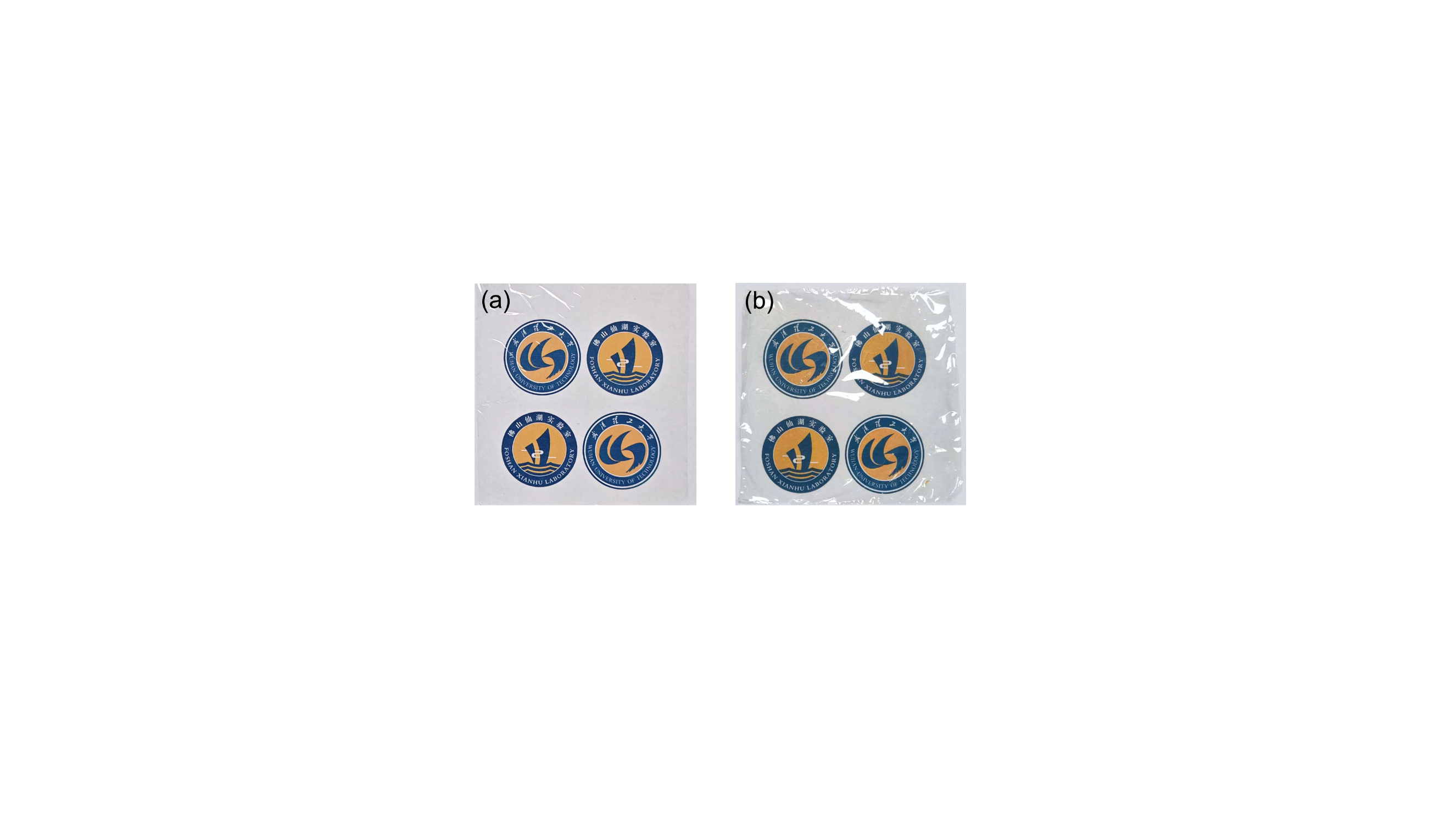


**Figure S2**. The optical photographs of (a) PFSI-PA and (b) PFTSI membrane.


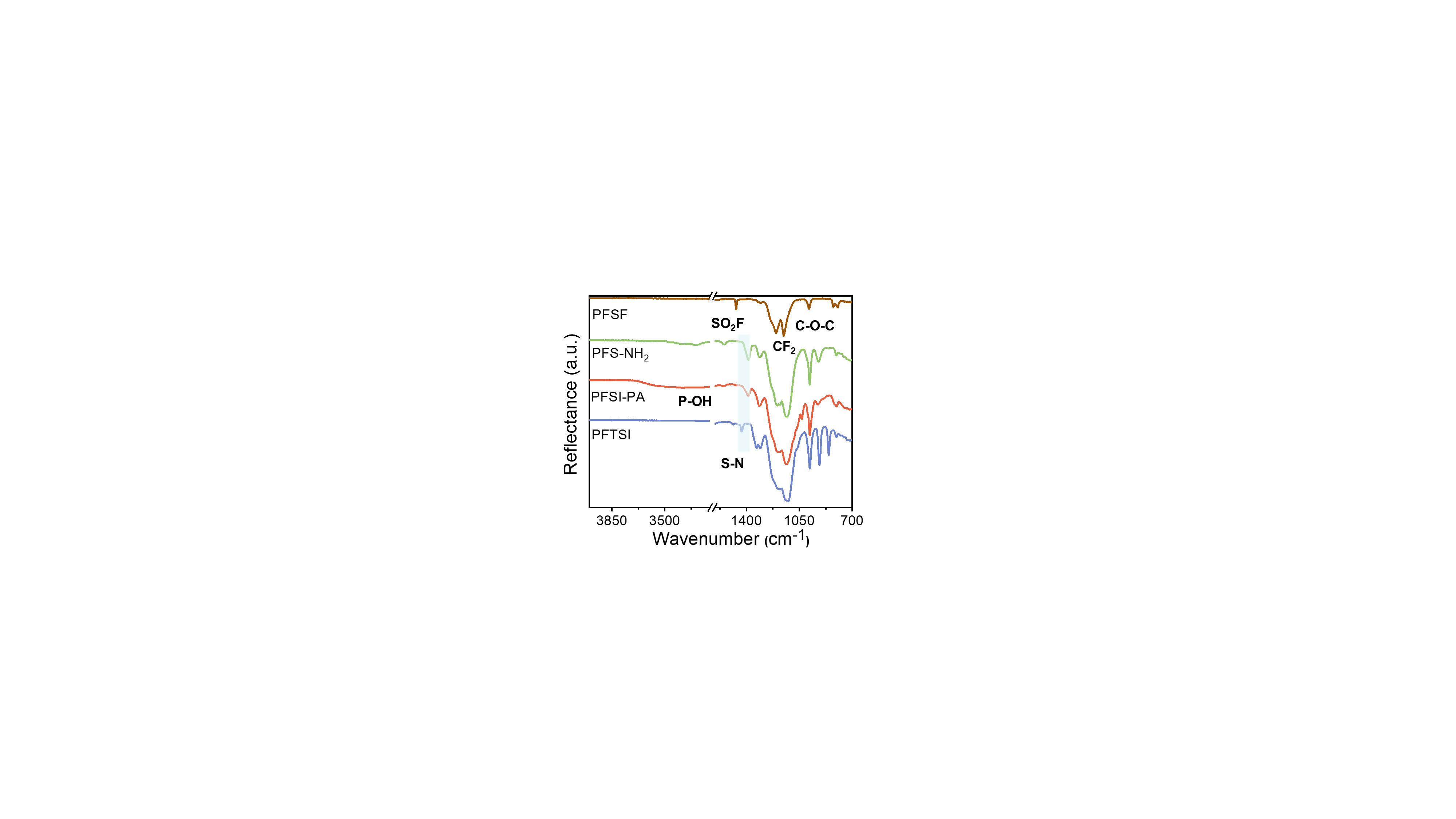


**Figure S3**. ATR-FTIR spectrum of the PFSF, PFS-NH_2_, PFSI-PA and PFTSI.


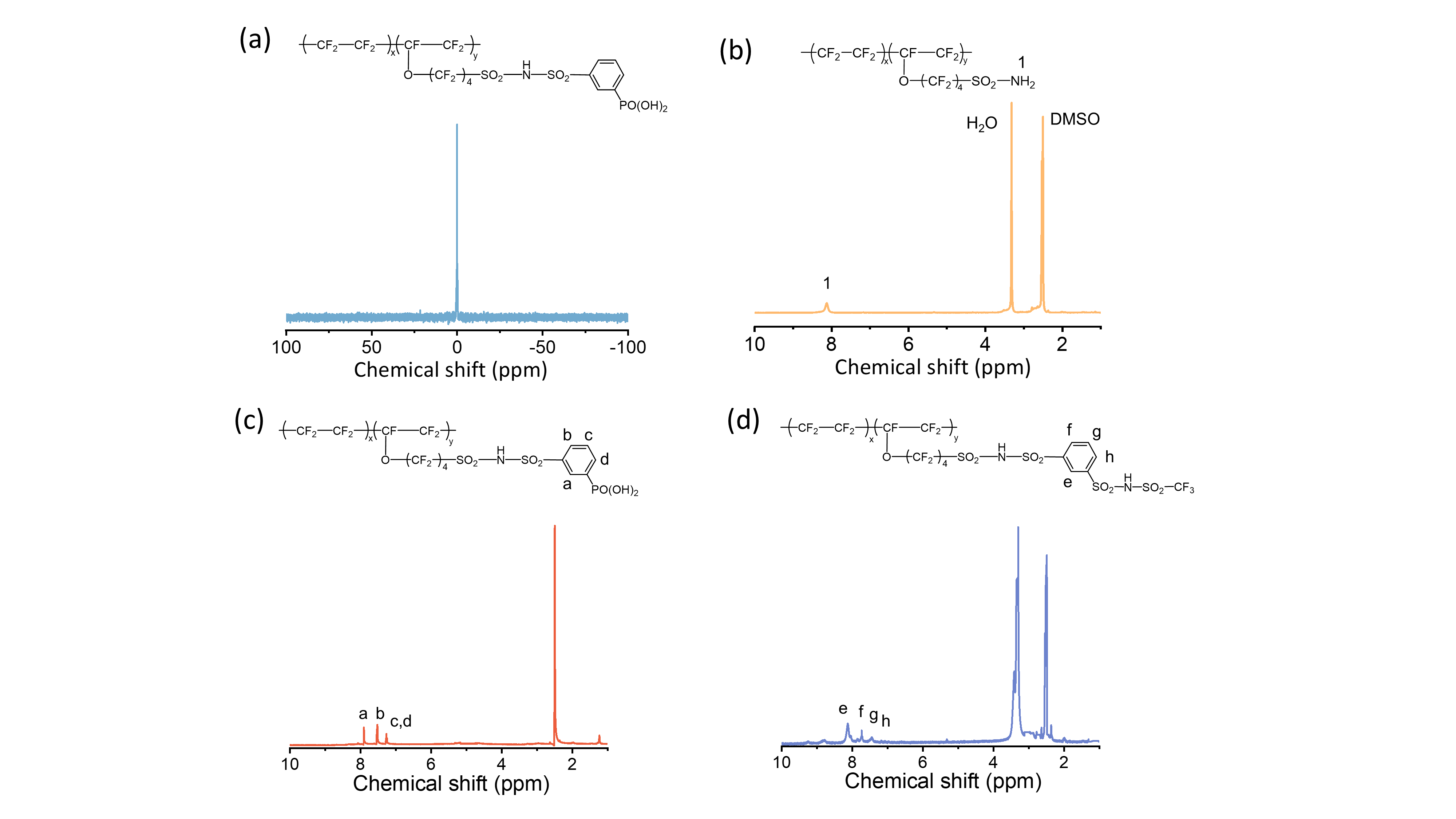


**Figure S4**. (a) The ^31^P Nuclear magnetic resonance spectroscopy (NMR) spectrum of PFSI-PA. The ^31^H NMR spectrum of (b) PFSI-NH_2_, (c) PFSI-PA and (d) PFTSI.


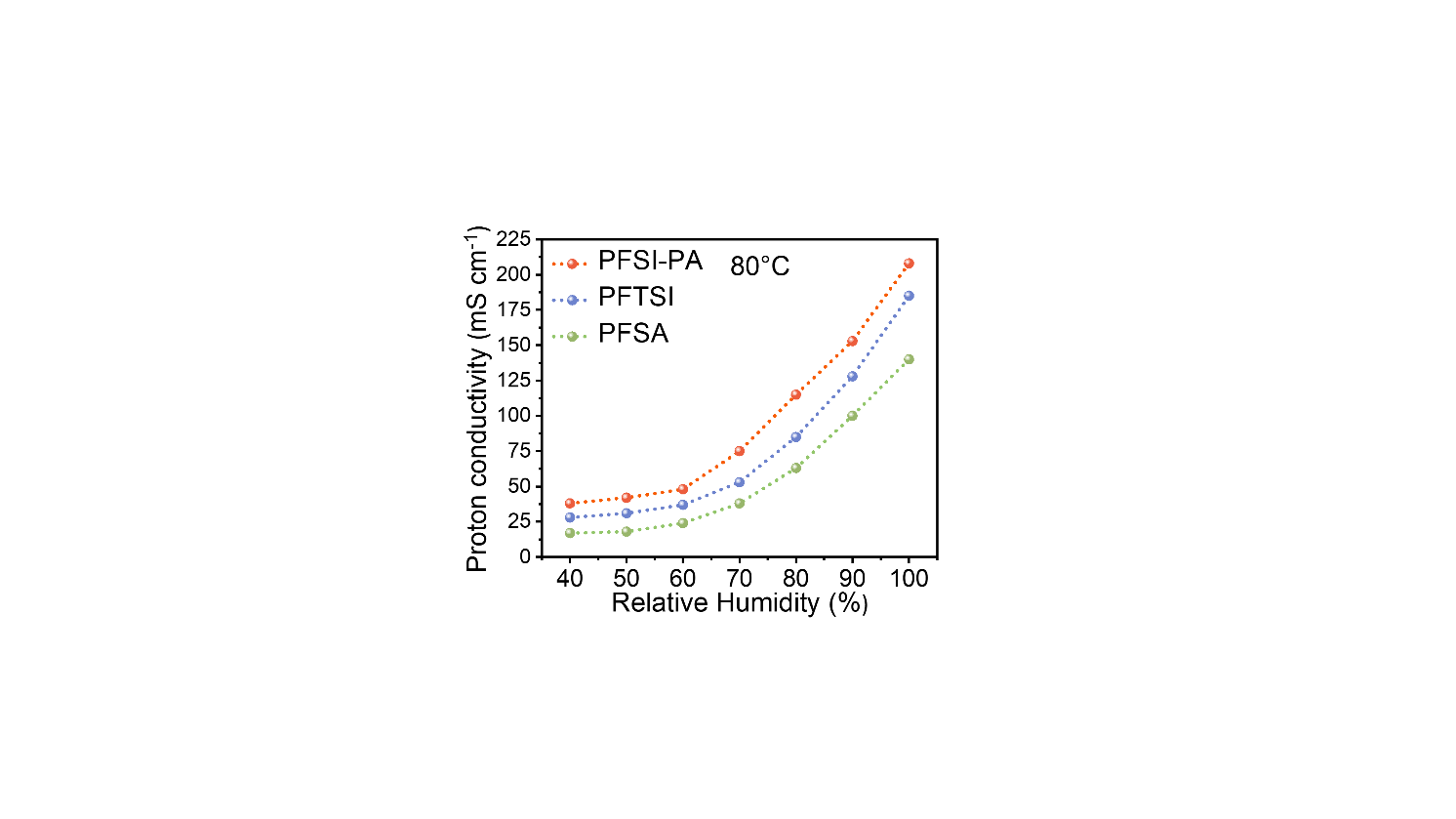


**Figure S5**. In-plane proton conductivity of PFSI-PA, PFTSI and PFSA membranes at 80°C.


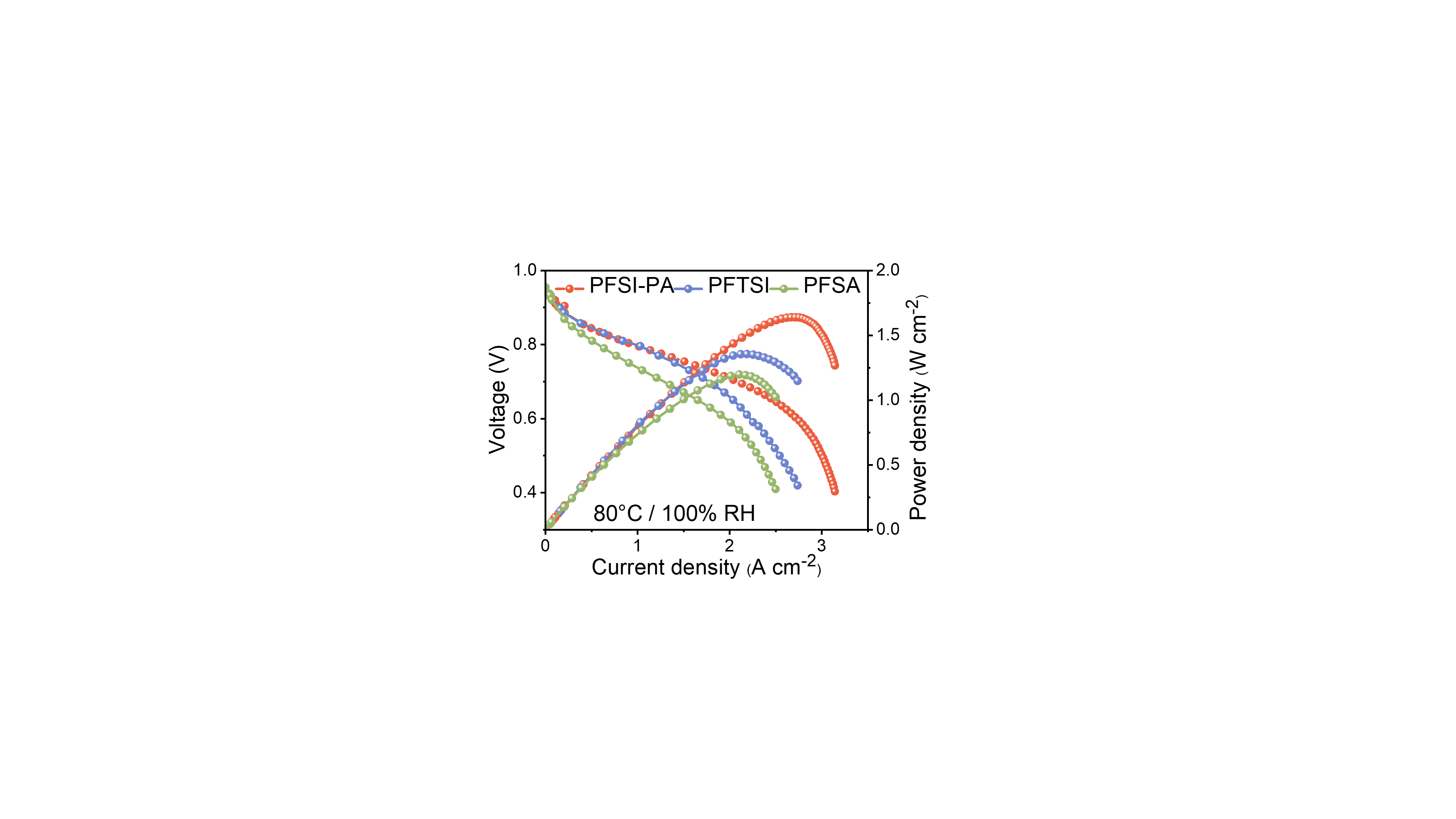


**Figure S6**. Polarization and power densities curves of PFSI-PA under 100%RH.


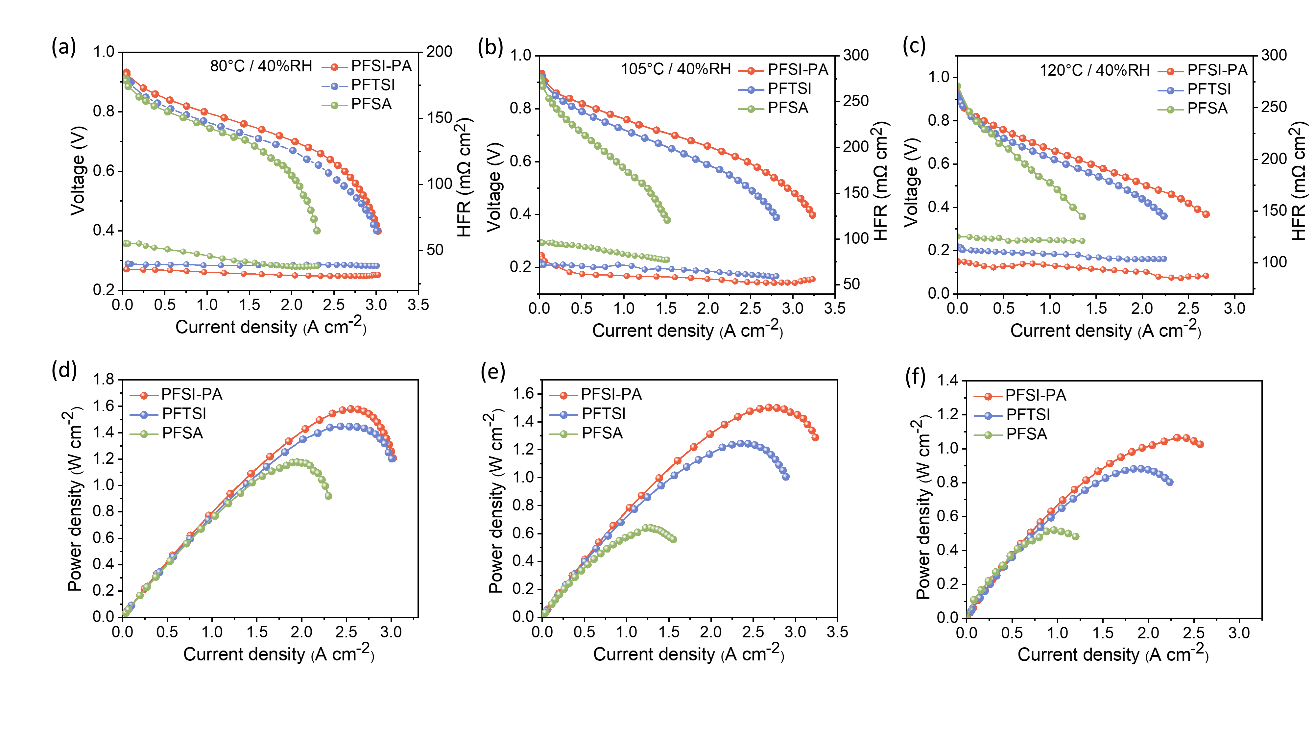


**Figure S7**. Polarization / high-frequency resistance (HFR) and power densities curves of as-prepared MEAs under different test conditions: (a, d) 80°C/40%RH, (b, e) 105°C/40%RH and (c, f) 120°C/40%RH.


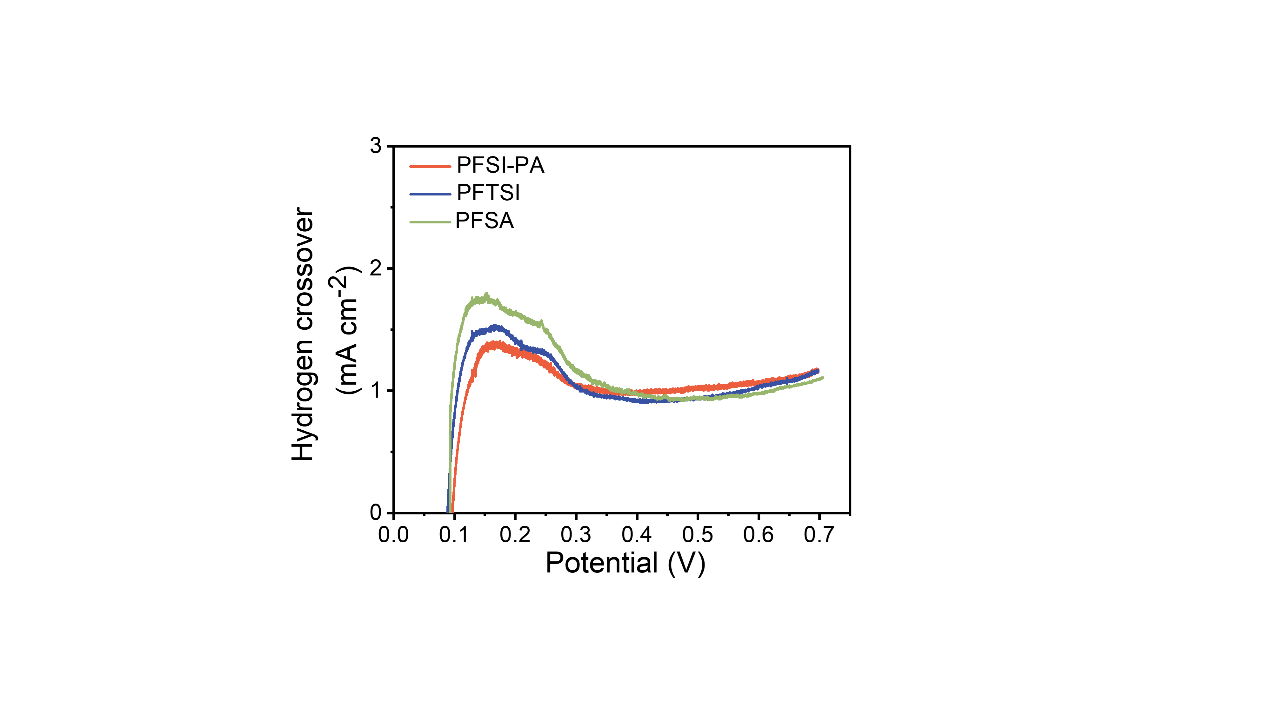


**Figure S8**. Hydrogen crossover results of the prepared MEAs.


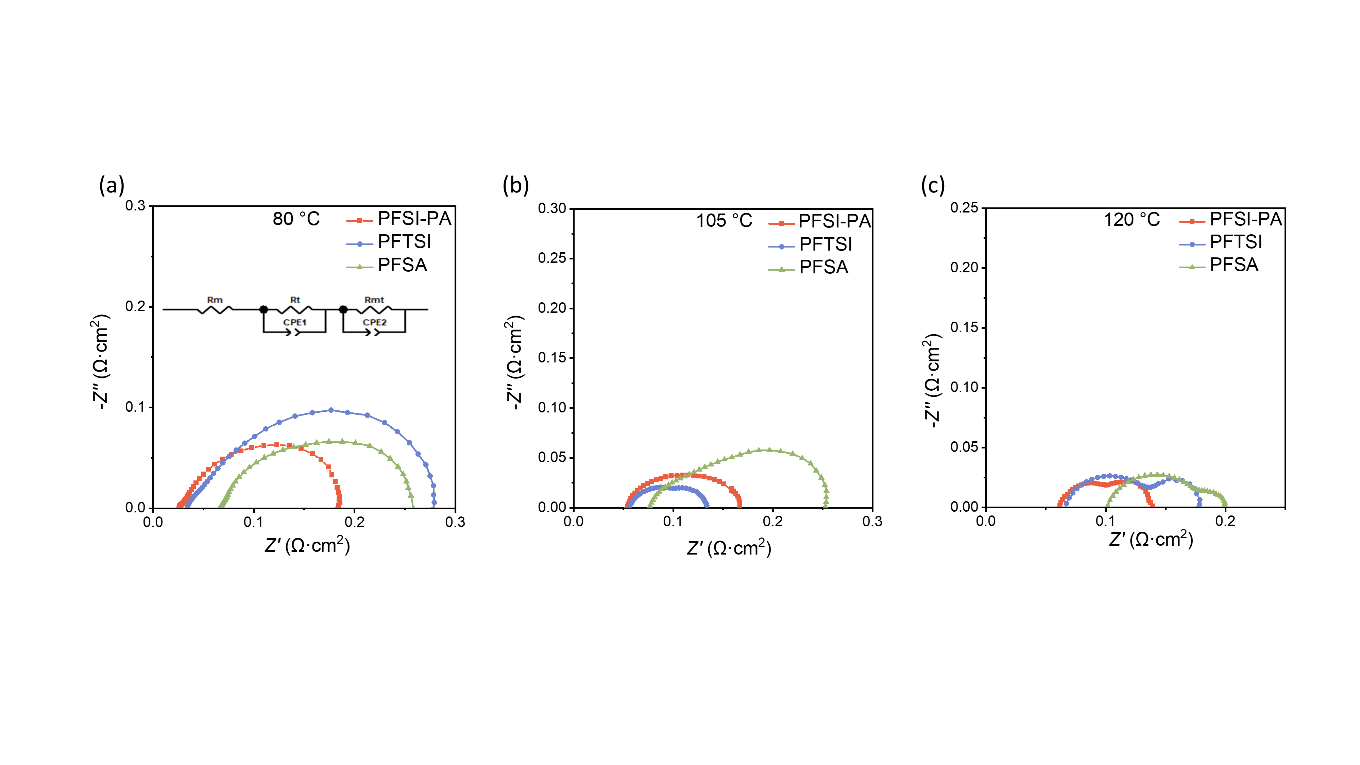


**Figure S9.** Nyquist curves under 0.65 V at (a) 80℃ (the inner figure shows the equivalent circuit diagram model), (b) 105℃ and (c) 120℃.


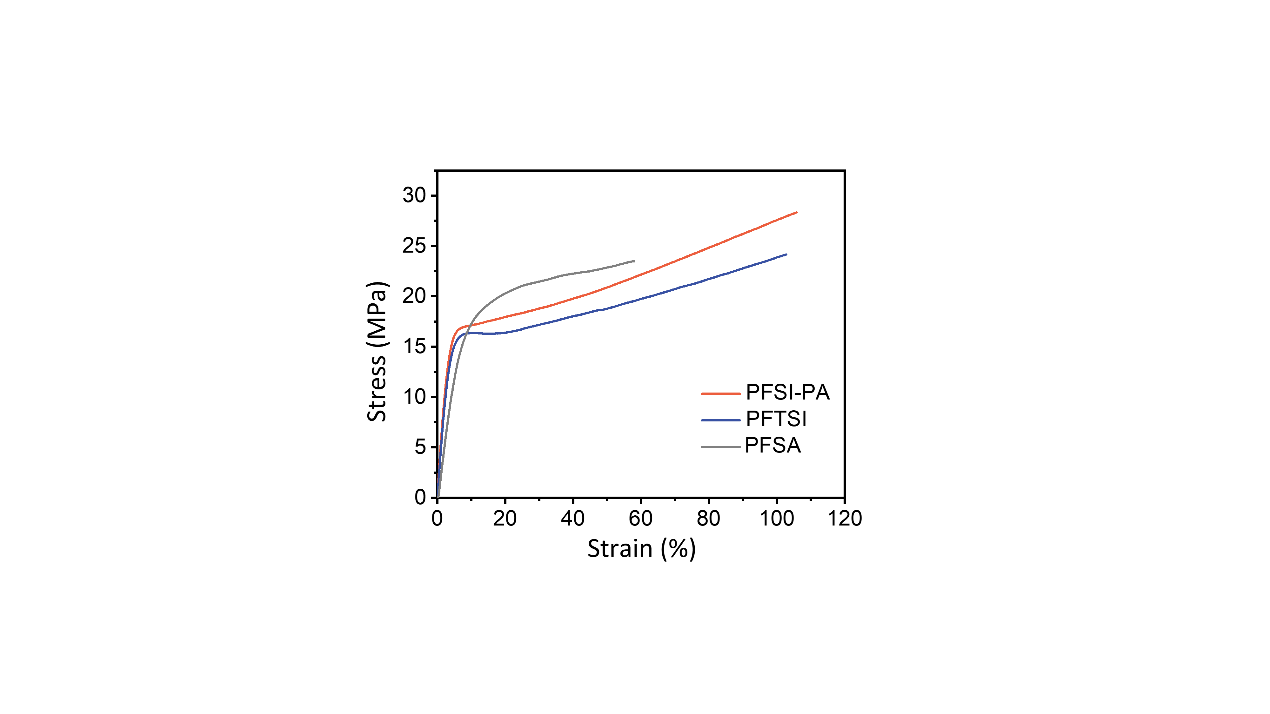


**Figure S10**. The stress *versus* strain curves of the prepared membranes.


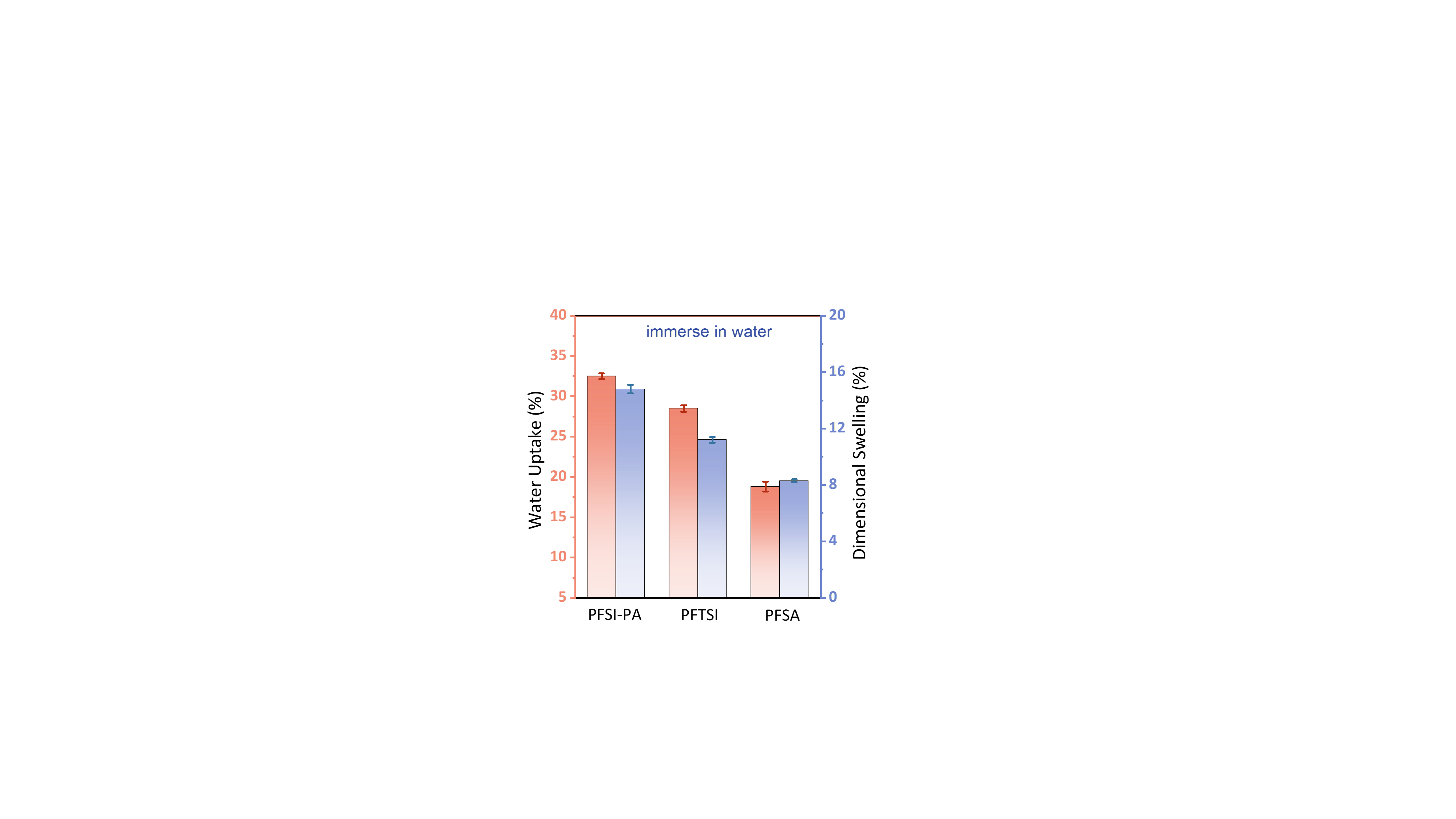


**Figure S11**. The Water uptake and Dimensional swelling of PFSI-PA, PFTSI, and PFSA membranes.


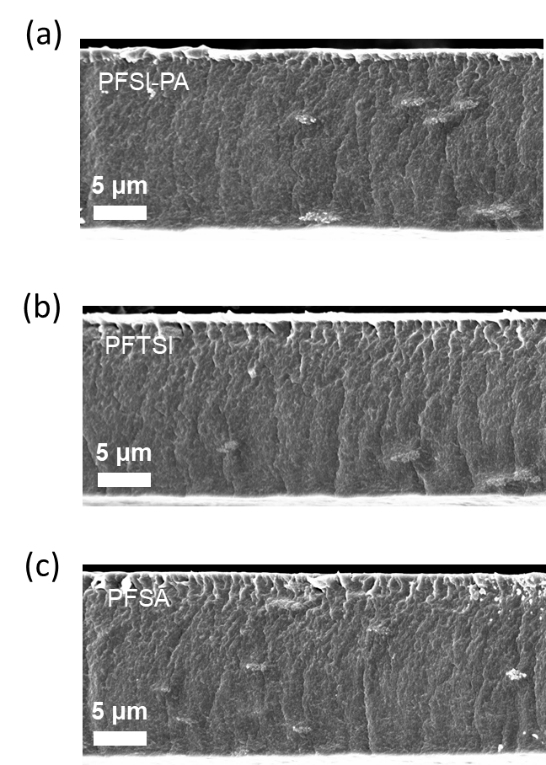


**Figure S12**. (a, b and c) the cross-section SEM images of PFSI-PA, PFTSI and PFSA.


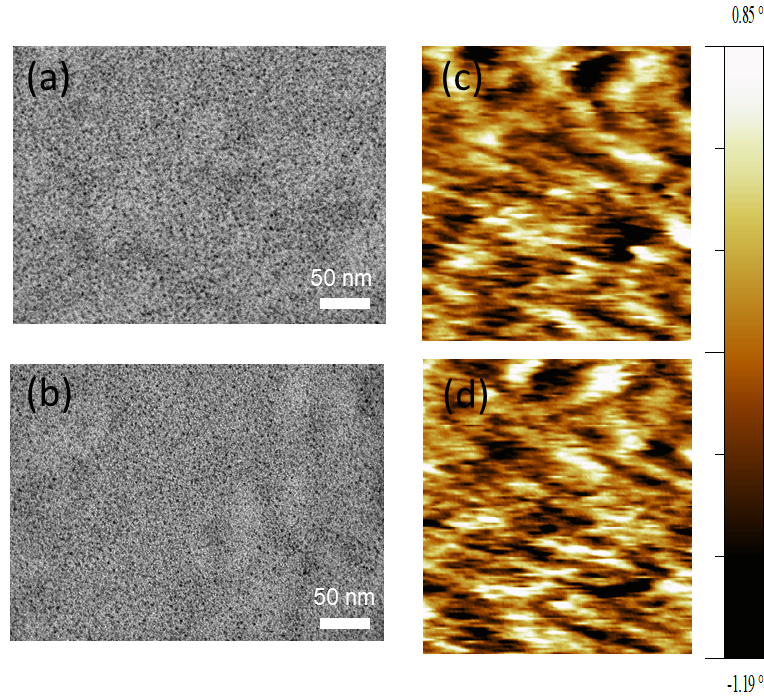


**Figure S13**. The TEM images of (a) PFSI-PA and (b) PFTSI. The AFM phase images of (c) PFSI-PA and (d) PFTSI.


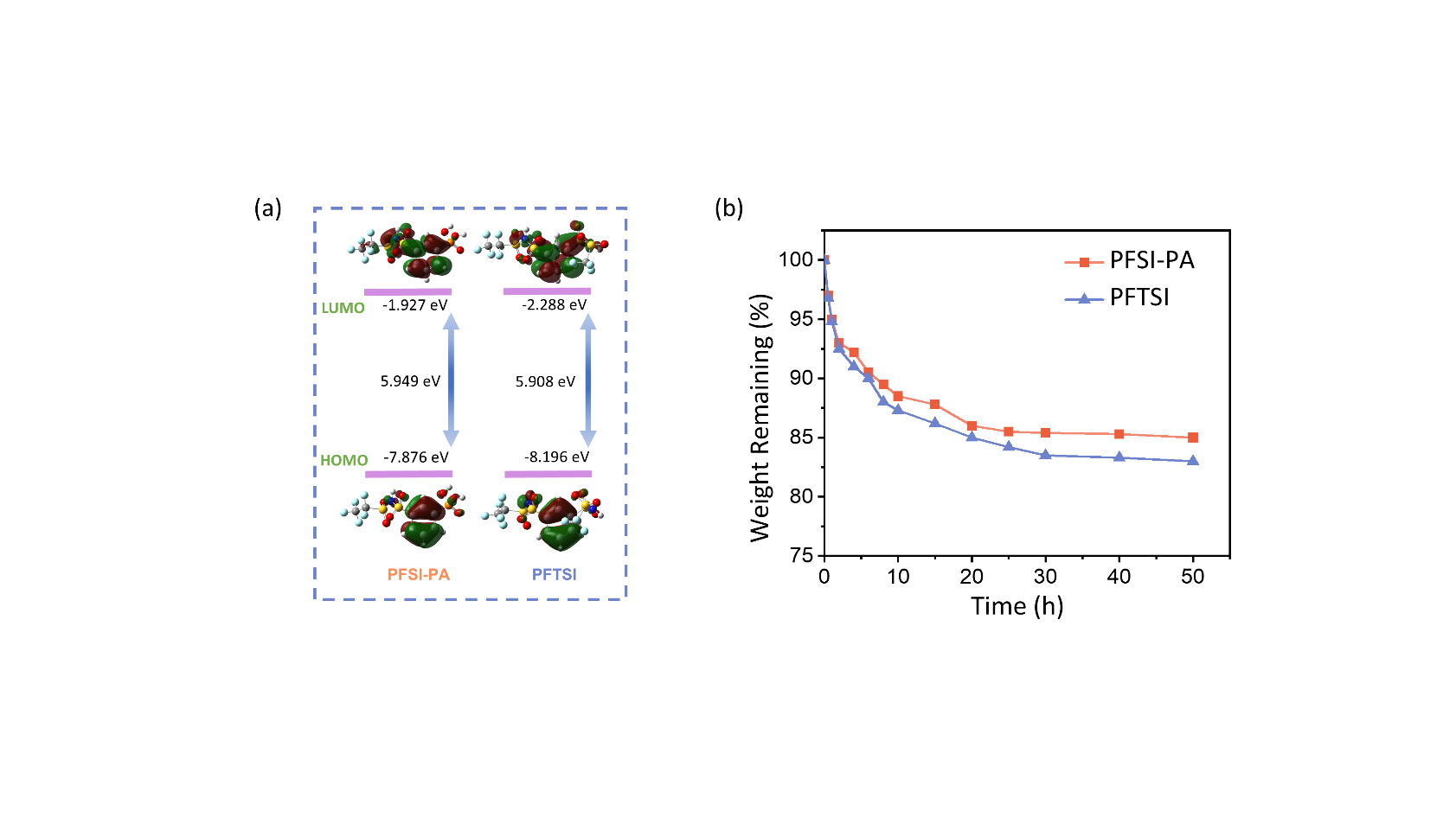


**Figure S14**. (a) Lowest unoccupied molecular orbital−highest occupied molecular orbital (LUMO−HOMO) plot and the energy level of the PFSI-PA and PFTSI. (b) Weight Remaining after Fenton test of PFSI-PA and PFTSI membranes.


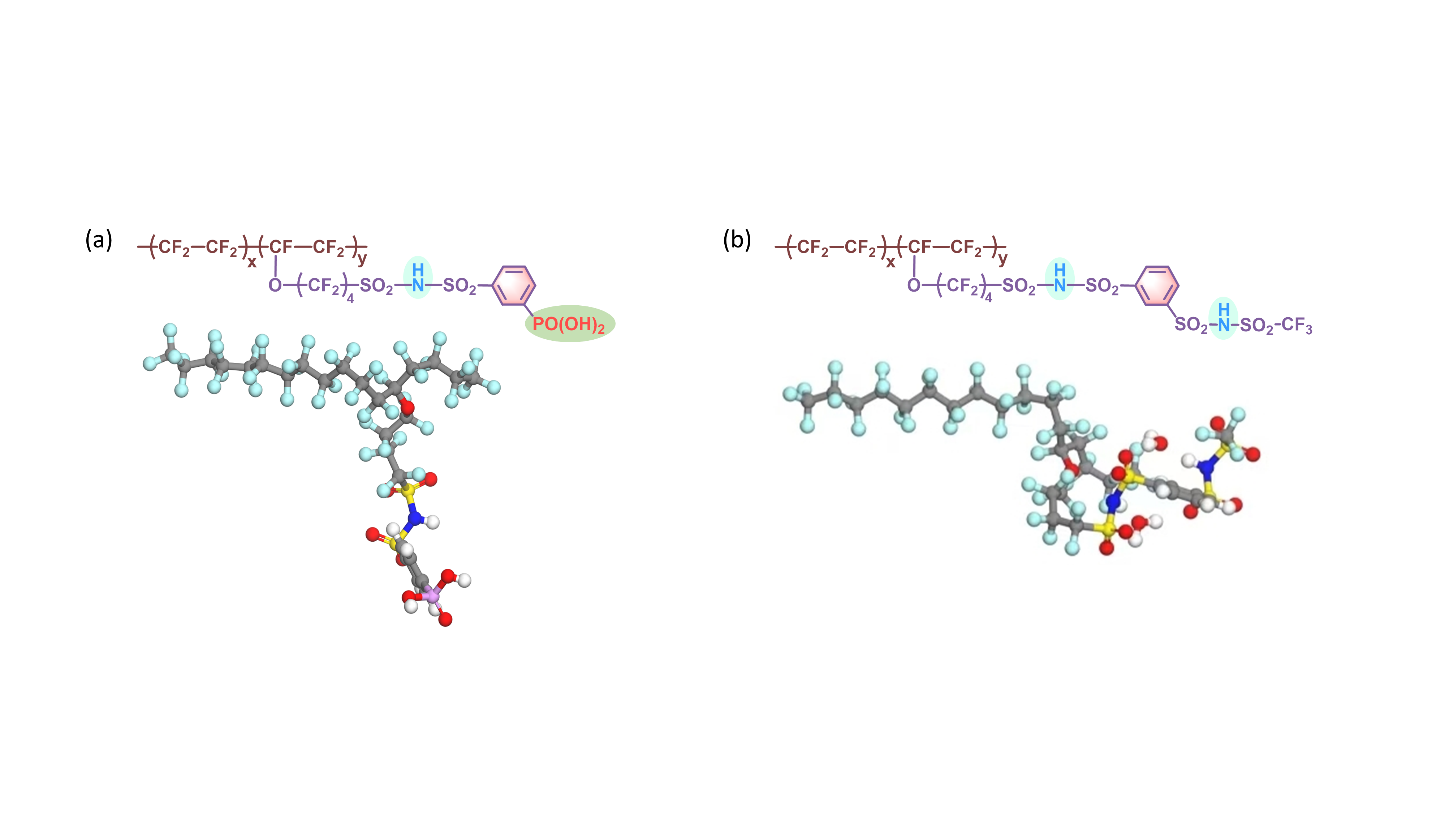


**Figure S15**. Molecular structure and schematic illustration of the coarse-grained model of (a) PFSI-PA and (b) PFTSI.


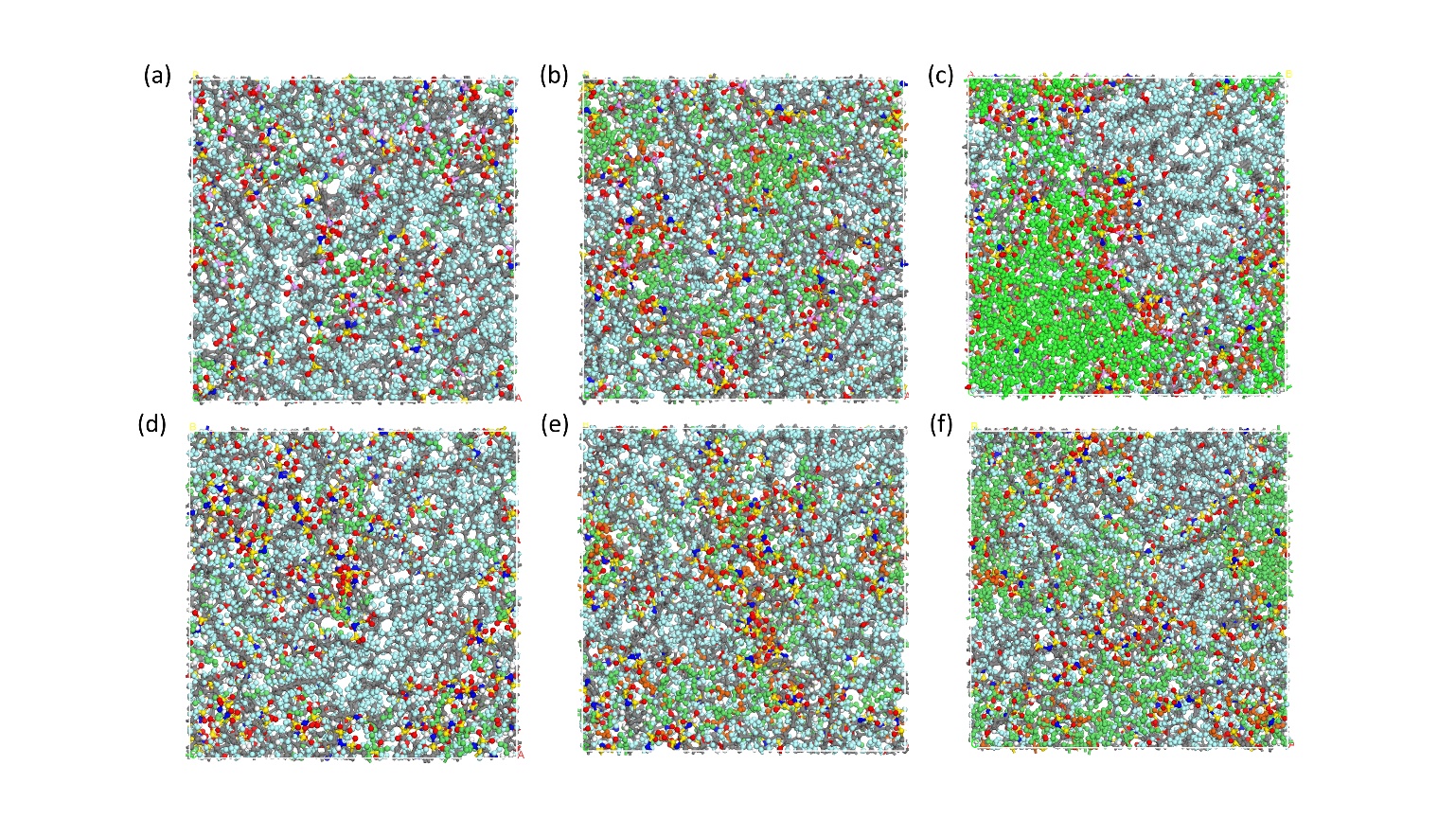


**Figure S16**. Simulated snapshots of each are extracted from the entire system at different hydration levels for (a, b and c) PFSI-PA and (d, e and f) PFTSI.

.


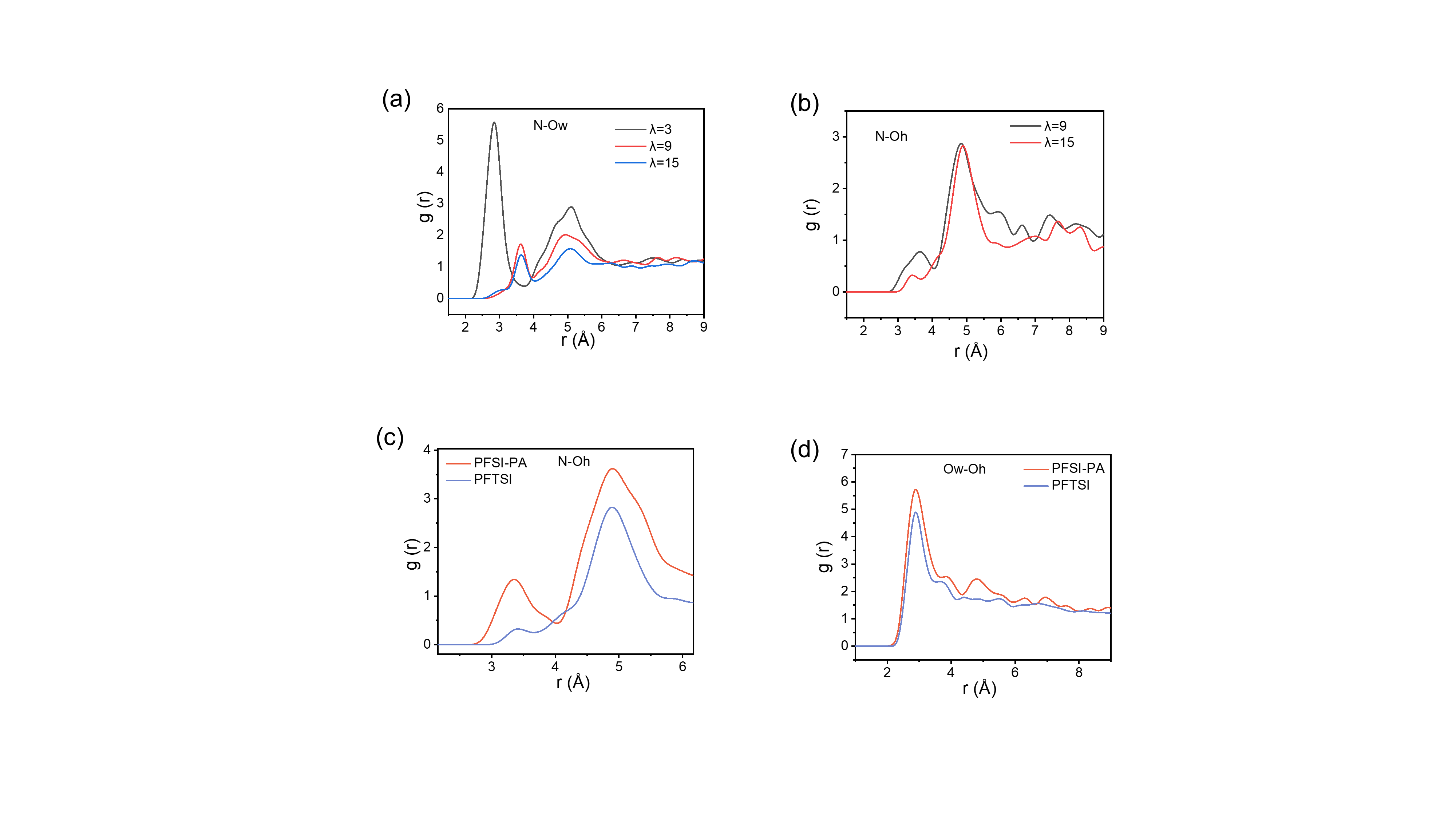


**Figure S17**. PFTSI RDFs at different hydration levels for (a) N-Ow, (b) N-Oh. PFSI-PA and PFTSI RDFs at λ=15 for (c) N-Ow and (d) Ow-Oh.


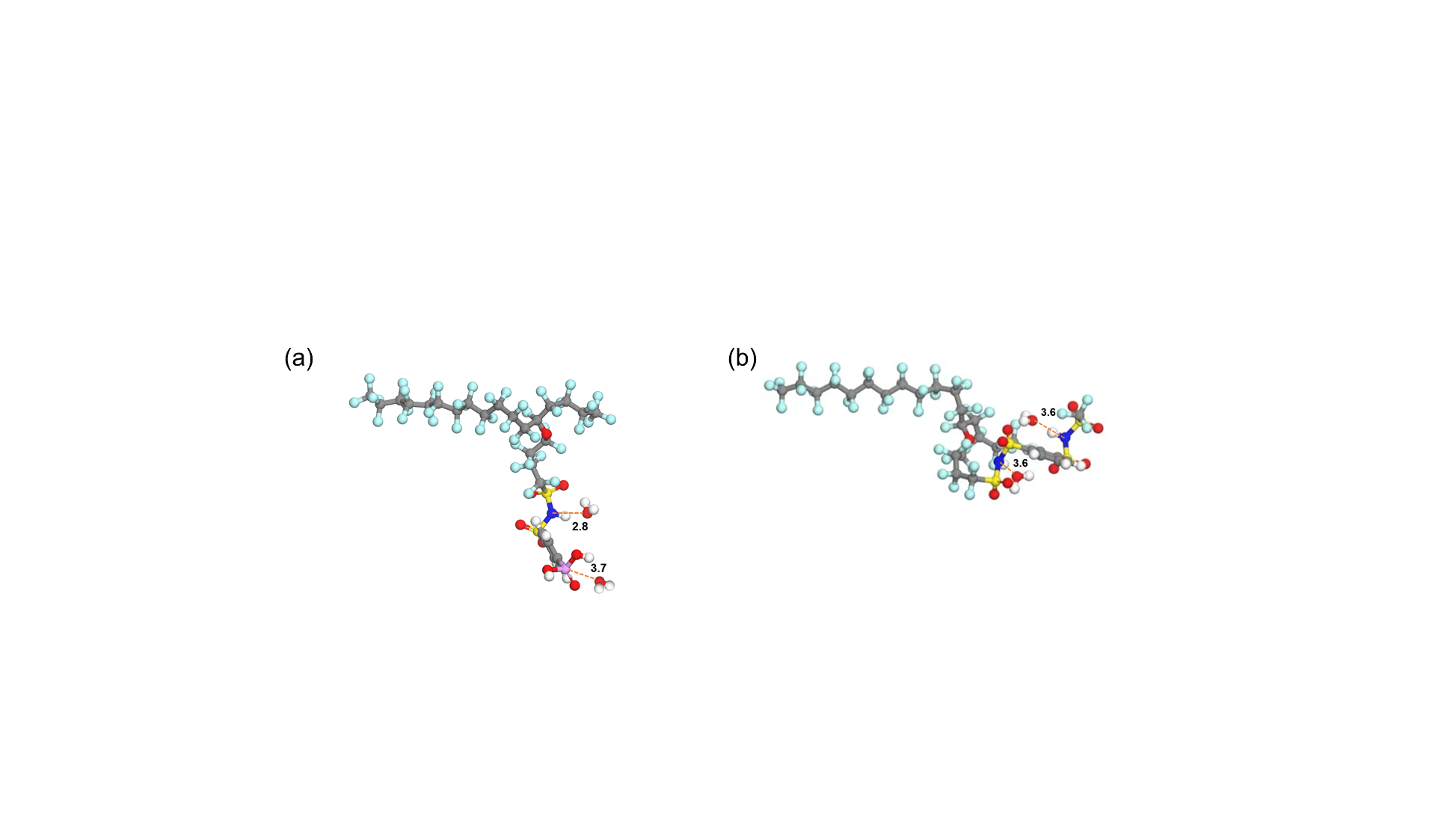


**Figure S18**. Schematic diagram of the spatial distance between water molecule and acid groups in (a) PFSI-PA and (a) PFTSI.


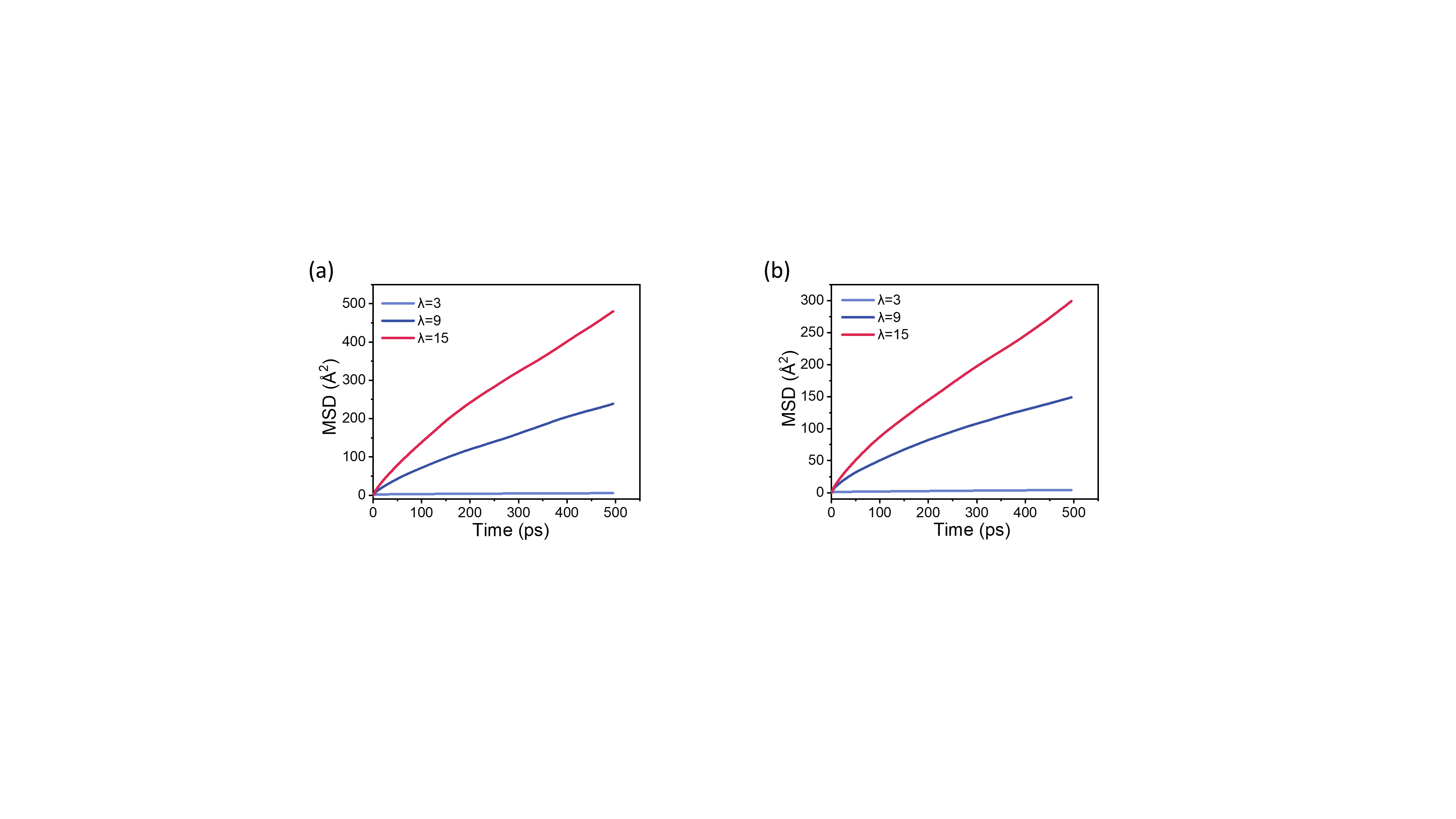


**Figure S19**. MSDs for hydronium ions at different hydration levels of (a) PFSI-PA and (b) PFTSI.

1. [↑](#footnote-ref-1)
